# Supplementary material for: Discovery of key whole-brain transitions and dynamics during human wakefulness and non-REM sleep
Source: Nat Commun. 2019 Mar 4;10:1035. doi: 10.1038/s41467-019-08934-3 (PMC6399232; doi:10.1038/s41467-019-08934-3)
Supplement: Supplementary file 1 — Supplementary Information [file 41467_2019_8934_MOESM1_ESM.pdf]

Supplementary Information for the manuscript:

Discovery of key whole-brain transitions and dynamics during human wakefulness and non-REM sleep

Stevner, A.B.A. et al.

Supplementary Discussion 1

The HMM was sensitive to recurrent changes in both mean BOLD activity and FC, and the overall reflection of PSG stages in the whole-brain network states, seen also form the MANOVA results, suggests that these two features of brain activity changed reliably with PSG stages. This is in line with previous PET [1, 2, 3, 4] and fMRI [5, 6, 7] studies that have regressed PSG stages onto neuroimaging data to show differences between individual PSG stages in metabolic and BOLD activity, an EEG study showing PSG-dependent changes in EEG microstates [8], demonstrations that machine-learning can be used to classify fMRI recordings into PSG stages based only on FC patterns [9, 10, 11], and most recently the study by Haimovici and colleagues demonstrated that PSG stages could be identified as individual states of dynamic FC [12].

We found high-order RSNs, i.e. the DMN and the ACN, to occur relatively exclusively during wakefulness. However, previous investigations have suggested a rather ubiquitous presence of both the DMN and the ACN, not just in wakefulness but, in all stages of NREM sleep [6, 13, 14, 15]. These studies were based on static FC analyses of PSG-defined sleep stages, using both seed-based correlations [6, 13, 14, 15] and spatial ICA [16], and the seeming lack of sensitivity to changes in vigilance has raised questions about the relevance of these RSNs for on-going cognition [17]. Our results contribute to this debate, by suggesting a clear wakefulness-specificity of the DMN and the ACN in terms of mean activation, but not in FC (see also Supplementary Note 5).

Two HMM states were clearly specific to periods of N2 sleep. The corresponding mean activation maps showed either increases or decreases in areas consistently identified in a number of studies as fMRI-correlates of sleep spindles [18, 19, 20]. We tested the temporal relationships between the data-driven HMM states and the occurrence of sleep spindles, as identified in the EEG (see Supplementary Figures 19–20 and Supplementary Note 4), and indeed we found HMM states 3 and 6 to account for the majority of time where sleep spindles were present. However, the same was true for K-complexes. When identifying K-complexes from the EEG and comparing their occurrences with the HMM states, we once again found HMM states 3 and 6 accounting for the majority (see Supplementary Figures 19 and 21). In line with previous studies investigating the effects of spindles and K-complexes on the BOLD signal [18, 20, 21] we also looked at the temporal relationships to the HMM states after convolving the sleep graphoelements with the canonical hemodynamic response function (HRF, see Methods and Supplementary Figure 19 for illustration).

This led to an increase in the correlation and specificity values of the HMM states compared to when the raw non-convolved occurrences of spindles and K-complexes were used, but overall did not change the differences on these scores between the states. HMM states 3 and 6 still accounted for the majority of the HRF-convolved sleep spindles and K-complexes. Within HMM states 3 and 6 we did not find marked differences in their relationship to the graphoelements. Both with and without the HRF convolution HMM state 3 tended to show higher correlation and specificity values than HMM state 6 for both spindles and K-complexes, although these differences did not survive correction for multiple comparisons, as shown in Supplementary Figures 20 and 21. The theoretical scenario that the two HMM states (3 and 6), accounting for the majority of N2 sleep, might represent direct reflections of different sleep graphoelements does thus not seem to be supported. Rather, both of these HMM states included each of their share of both spindles and K-complexes. In light of previous event-related demonstrations of robust effects of sleep graphoelements on the BOLD signal [18, 19, 20, 21, 22], as well as the similarity between these event-related patterns and the mean activation maps of HMM states 3 and 6, it seems unlikely that spindles and K-complexes did not influence the HMM state description, however this did not result in any HMM state coding exclusively for one or the other graphoelement. There is of course the possibility that HMM states 3 and 6 are suggesting a categorisation of spindles and K-complexes beyond what the scalp EEG is able to resolve, or at least beyond the classical interpretation of these graphoelements. Future mapping of the spectral and spatial properties of these graphoelements at higher resolutions than the AASM criteria, that were used here, could bring more insights in this regard, as could a combination or comparison with intracortical evidence [23, 24, 25].

There is growing evidence that neuroimaging timecourses contain long-range temporal dependencies [26, 27, 28], i.e. they are non-Markovian [29]. The HMM used here follows the Markovian assumption in the sense that the probability of a state transition at a given time point depends only on the state that is active at the preceding time point, and hence it does not parametrically model long-range temporal dependencies. Importantly, however, it does not preclude them either. This means that the HMM state timecourses can in fact exhibit non-Markovian dynamics and long-term dependencies; see e.g. [30]. Notably, our finding of HMM states grouping into modules of transitions represents an analysis that goes beyond Markovianity, and demonstrates non-Markovian dynamics (i.e. long-term dependencies) at the system level of the HMM states. In light of this, our finding that N3 sleep was modelled almost exclusively by a single HMM state, while several states grouped into modules during wakefulness, is in line with the study by Tagliazucchi and colleagues, showing that long-range temporal dependencies in fMRI signals decreases from wakefulness to N3 sleep [16].

Supplementary Discussion 2

Methodological considerations

For the HMM analysis we chose to make use of the full dataset of 57 participants, when inferring the states. Subsequently we analysed the part of the HMM solution that corresponded to the 18 participants that included all PSG stages. We chose to include as much data as possible for the initial inference in order to maximise the signal-to-noise ratio and amount of evidence for the HMM parameter estimation. Yet, as it is clear from Supplementary Table 1, the full dataset included rather uneven distributions of PSG stages. While PSG stages are more evenly distributed in the subset of 18 participants, and we diligently made sure to normalise the relevant summary measures by number of samples of PSG stages within participants, there still exists a possibility that the HMM could be biased by having more data available from certain PSG stages than others, even if the HMM remained uninformed of the PSG staging. For instance, one could imagine that more data from a certain PSG stage would lead to more HMM states per time being assigned to data from that PSG stage. Such an effect is not immediately present in our results, however, since for instance we found both switching and range of HMM states to be higher in N1 sleep, even though the original full dataset included more than twice as much data from wakefulness (see Supplementary Table 1 and Figure 3d–e).

The number of HMM states was set to 19 in our analyses based on an evaluation of a range of HMM solutions with varying numbers of states (see Methods). It is important to note, however, that it is difficult to determine a 'correct' number of states, when decomposing continuous recordings of brain activity. The recent study by Haimovici and colleagues aimed to identify individual FC states for each PSG stage, and thus chose 4 states for their sliding-window analysis [12]. Our aim was to extract as much temporal resolution as possible from the BOLD ROI timecourses. Ideally, a higher number of states should provide more temporal detail, however increasing the number of states above 19 was associated with a higher occurrence of 'sporadic' HMM states, modelling very specific subparts of the data and not generalising across participants (see Methods and Supplementary Note 3). Based on this it is important to emphasise that we do not suggest the number 19 as definitive, but simply a tool to resolve as much temporal information as possible from the current dataset.

It should be noted that the HMM framework was chosen over other methods for extracting dynamic states from multivariate neuroimaging datasets, such as sliding-window clustering [12, 31, 32], point-

process analysis [33], and co-activation pattern analysis [34, 35] (for reviews, see [36, 37]). The employed HMM framework has been successfully applied to resting state data of wakefulness in both MEG [38, 39, 40] and fMRI [30, 41], and was particularly suitable for our purpose by virtue of its explicit modelling of temporal dynamics, resulting in states that repeat in a predictable way. Although the HMM is not a mechanistic model of brain activity (a limitation shared with the alternative approaches mentioned above) we have shown how the explicitly modelled HMM transition matrix was fundamental to suggest new partitions of dynamic whole-brain states, which future mechanistic frameworks of NREM sleep and wakefulness should take into account [42, 43, 44, 45].

Sleep is of course a process associated with profound physiological changes, not merely those reflected in brain activity [46]. Despite our use of the RETROICOR method to reduce the effects on the fMRI data of cardiac and respiratory signals (see Methods), it is currently not possible to completely isolate the neural effects of sleep in fMRI. Our results hence share the limitation with other neuroimaging studies of sleep of potentially being influenced by physiological changes not directly linked to brain activity. On the other hand, certain sleep-dependent peripheral changes such as those of the autonomic nervous system will also induce genuine activities in the brain, which in future studies would be important to investigate and with the proper recordings could potentially be evaluated within an HMM framework.

In addition, it should be noted that there could be potentially confounding effects of spatially smoothing the fMRI data, which can create artificial dependencies between regions of interest. In the context of the HMM, which focuses on the aspects of the data that represent more variance, this confound is however likely to be minor.

Another potential caveat of our analyses pertains to the initialisation of the HMM, which is not deterministic. In Supplementary Note 2, we provide a summary analysis showing that the HMM infers consistent states across independent initialisations and splits of data (see Supplementary Figure 16)

Supplementary Discussion 3

Perspectives

Features identified by the HMM could prove to be essential supplements to PSG and other conventional methods when trying to understand phenomena like the subjective perception of sleep [47, 48], mental content during sleep [49, 50], such as the hypnagogic or even hallucinogenic character of

sleep onset [51, 52], sleep inertia of the awakening process [53], sleep-dependent processes related to memory and learning [54], and disordered sleep, like insomnia [55]. Such studies should explore the theoretical potential of applying the current HMM, parameterised on the present sleep fMRI data, to identify the presence of the same dynamical whole-brain network states and transition modules in data from different cohorts, potentially even at the individual level. This new data could then be linked to behaviour and cognition through sophisticated measures of arousal, such as eyelid-closure [56], sleep mentation [49], post-sleep memory- and learning performance [57], and careful clinical examination of sleep disorders [58, 59, 60].

The wake-NREM sleep cycle merely represents a sub-part of a continuum of activities that the brain supports. Other important brain processes should be sought integrated with the presented transition map (Figure 7), most obviously including REM sleep, but also other altered states of consciousness, such as anaesthesia [61, 62, 63], the psychedelic experience [64], and even different contents of consciousness during wakefulness [65].

Finally, there is scope for an even more detailed examination of sleep within the HMM framework, given that BOLD data is not the most temporally sensitive modality available. Recently developed methods combining the HMM framework with source-reconstructed MEG data could prove capable of providing an even more fine-grained picture of sleep's evolution in whole-brain networks, and allow for an examination of microstructural EEG elements of sleep, such as spindles and K-complexes [38, 39, 40, 66], as well as EEG-markers of vigilance fluctuations during wakefulness [67].

Supplementary Note 1

Robustness across different parcellations

We chose the AAL over other possible parcellations because it is the most frequently used in previous fMRI studies of FC during NREM sleep [15, 16, 17, 18, 19, 20]. Alternative parcellations, such as those derived from FC configurations in the data, could be problematic, since FC has been shown to robustly vary across the sleep cycle [9, 10, 11]. Being anatomically defined, the AAL is essentially agnostic to potentially changing FC configurations within the data. In order to make sure that the use of the HMM generalises to different levels of spatial granularity and that the interpretation following from our results were not specific to the use of the AAL atlas, we re-ran the HMM with a different parcellation. While the field of proposed parcellations for large-scale neuroimaging is rapidly expanding [68], we opted for the Brainnetome atlas, originally published by Fan and colleagues [69]. Unlike many of the most popular parcellation schemes, the Brainnetome is not solely

derived from fMRI FC, but also depends on structural connectivity information for its partitioning of the brain volume. As mentioned above, the use of an FC-derived atlas could bias results, since FC has a well-established dependence on vigilance. Another advantage of the Brainnetome is that it, like the AAL, includes sub-cortical regions, which, as shown in the Results and Discussion of the main text, undergo important changes in activity across NREM sleep. Finally, the 246 regions of the Brainnetome atlas compared to the 90 regions of the AAL provides a good test for the robustness of the HMM across different levels of spatial granularity.

We followed exactly the same steps as explained in the Methods section, but extracted ROI timecourses from the Brainnetome atlas instead of the AAL. It became clear that the increase in spatial detail, going from the AAL to the Brainnetome, had an impact on the ability of the HMM to track the sleep scoring. As such, when using 90% of the variance from the PCA on the Brainnetome ROI timecourses (Figure 1), the performance of the HMM, as quantified through MANOVA between the resulting HMM state timecourses and the sleep scoring, was inferior to the original results using the AAL (see Supplementary Figure 22). However, a slightly stronger regularisation of the ROI timecourses, using only 85% of the variance from the PCA, made the results from the Brainnetome highly comparable to the original results using the AAL. At 85% of the variance the HMM on the Brainnetome data performed in a very similar fashion to the HMM on the 90% of the AAL data, in terms of MANOVA and the development of median fractional occupancy across number of HMM states (see Supplementary Figure 22b and c). The difference between using 90% and 85% of the variance was importantly also evident in the number of HMM states that were consistent across participants for a given HMM solution (K = 19, see Supplementary Figure 22d). For 90% of the variance, only 6 HMM states occurred in more than 25% of the participants, whereas this number increased to 12, when 85% of the variance was used. In Supplementary Figure 23 we have re-constructed Figure 3 of the main text, but with the results using 19 HMM states on 85% of the variance of the Brainnetome data. The results are highly consistent, with individual HMM states showing sensitivity and specificity to different sleep stages, and in terms of the differences in dynamics found between sleep stages. Regarding the spatial configuration of the HMM states resulting from the Brainnetome data, these were also highly consistent with the original HMM states using the AAL. This is illustrated in Supplementary Figures 24 and 25, where we have matched HMM states from the Brainnetome to the original HMM states, based on their specificity profiles to sleep stages and spatial patterns.

Overall, the above analysis shows that increasing the spatial granularity by introducing a different parcellation comes at the cost at decreasing the signal-to-noise ratio on the HMM estimation. However when this is controlled through PCA, results can be brought to convergence.

Supplementary Note 2

Robustness across different HMM initialisations

The initialisation of the HMM includes a stochastic element. To make sure that the states inferred by the HMM were not contingent on the initialisation, we ran the HMM with 19 states an additional four times on the full dataset ($N = 57$), and five times on each of the two half-splits of the data ($N = 29$ and $N = 28$). The 19 resulting states of each HMM repetition were matched to the states of the original HMM. Each state of a repetition was thus paired to an original HMM state, based on the similarity between their respective Gaussian distributions. The similarity was estimated using the Bhattacharyya distance [70], and the matching of states across repetitions were carried out using the Munkres algorithm [71].

Following the pairing of states, all resulting states were compared in an all-to-all manner, again using the Bhattacharyya distance as a measure of similarity. The resulting matrix $[(n_{\text{dataset}} + n_{\text{states}} + n_{\text{repetitions}}) \times (n_{\text{dataset}} + n_{\text{states}} + n_{\text{repetitions}})]$ is shown in Supplementary Figure 15a. The common pattern in the data-set-specific sub-matrices indicates that consistent HMM-state distributions were inferred across initialisation repetitions and data-splits.

Following the matching of the HMM Gaussian distributions from independent initialisations, we tested the temporal correspondence between the original HMM states and their counterparts from the repetition runs. This was done by comparing the corresponding state timecourses. For a pair of HMM states (one original and one from a repetition run) the temporal correspondence was quantified as the ratio between time points of overlap (simultaneous activity or inactivity) and time points of misses (off-sets of activity or inactivity). In Supplementary Figure 15b are plotted the mean values and standard deviations within data-splits, and it is clear that temporal overlaps outweighed misses for all runs of the HMM. This is an important indication that the evaluations of the HMM dynamics presented in the main text would be highly similar for other initialisations.

Supplementary Note 3

Varying the number of HMM states

In appreciation of the potential limitations related to choosing the number of HMM states with no strict, formal criterion, we include the results of using different numbers of HMM states. In Supplementary Figures 7 to 10 we have reproduced Figure 3 of the main text with HMM results using 15, 17, 21, and 23 states respectively. Demonstrating the robustness of our HMM findings,

the conclusions of the main text using 19 states are also found in Supplementary Figures 7 to 10. Specifically Supplementary Figures 7a-b to 10a-b show how select HMM states expressed high sensitivity and specificity for different PSG stages. In line with the results for K = 19 states, the HMM with lower and higher K identified states with high sensitivity and specificity for wakefulness, N2, and N3 sleep, but not for N1 sleep. Supplementary Figures 7d-e to 10d-e quantify the dynamics of HMM states within PSG stages. The relative differences between PSG stages are conserved and highly stable across numbers of HMM states. Interestingly, the absolute values of switching between and range of HMM states within PSG stages were in fact also quite preserved across numbers of HMM states. This is likely caused by the fact that the main effect of changing the number of HMM states is an addition of non-recurring, 'sporadic', states that modelled very (participant-) specific periods of the fMRI data (see Methods and Supplementary Discussion 2).

Another main result of this study is presented in the transition map of the HMM states (see Figure 4 of the main text). Again we have re-produced equivalent figures for K = 15, 17, 21, and 23 HMM states (Supplementary Figures 11 to 14). Our modularity analysis (see below) of the resulting transition matrices illustrates how the four modules from the HMM with 19 states can be identified in the solutions with different numbers of states. This was true for K = 17, 21, and 23. For K = 15, the white and blue modules appear to have merged together. The overall structure of the transition map was therefore robust across the chosen numbers of HMM states. A separate transition module for wakefulness after sleep onset (WASO) was found consistently across all of these values of K, while the intercalated module between wakefulness and consolidated sleep (N2/N3) were found for all but one value of K (K = 15).

Whereas these overall configurations of the HMM transitions were found robust to the chosen number of states, the more fine-grained details of the transition map appeared more variable. The gateway-like quality of a DMN-like configuration of brain activity was thus particularly clear for the originally chosen 19 states.

Supplementary Note 4

Relationship between HMM states and sleep graphoelements

In order to determine the effect of micro-structural features in the sleep EEG, (sleep graphoelements) on the HMM states we used information on the occurrence of sleep spindles and K-complexes during the fMRI recordings. The procedure for obtaining this information from the EEG for the present data has previously been described in Jahnke et al. [21]. Briefly, sleep graphoelements were manually identified according to the criteria set out in the AASM guidelines

[72]. This included the use of an EEG montage with frontal, central, and occipital electrodes re-referenced to the contra-lateral mastoid electrodes (TP9, TP10). The resulting temporal markings of sleep spindles and K-complexes were re-sampled to the sampling frequency of the fMRI acquisition (TR = 2.08 seconds) and collected in the variables SS-timecourse and KC-timecourse (for illustration of the SS- and KC-timecourse in an example participant, see Supplementary Figure 19). To account for the delay in the BOLD response, we also created versions of the SS- and KC-timecourses convoluted with the canonical hemodynamic response function (HRF). We used the HRF included in the SPM12 function spm_hrf.m (http://www.fil.ion.ucl.ac.uk/spm/). Specifically, SS-timecourse and KC-timecourse were binary and of the same length as the fMRI data, with ones representing the fMRI samples during which the respective graphoelement occurred, while the HRF-convolved versions were scaled between 0 and 1 with the canonical delays and undershoots (an example of the HRF-convolved timecourses is provided in Supplementary Figure 19c). We evaluated in turn the temporal association of each HMM activity timecourse to both sleep spindles and K-complexes. Three summary measures of association were used: i) Pearson's correlation was computed between each of the HMM state timecourses and the SS- and KC-timecourses within the set of participants that included the given graphoelement (see Supplementary Table 3 for an overview of the occurrence of sleep spindles and K-complexes). ii) Sensitivity to sleep spindles/K-complexes was quantified for each HMM state as the proportion of sleep spindles/K-complexes occurring during that given HMM state. iii) Specificity for sleep spindles/K-complexes was defined for each HMM state as the likelihood of finding that given HMM state active during an instance of the given graphoelement. The distributions across participants of these three summary measures are plotted in Supplementary Figures 20 (for sleep spindles) and 21 (for K-complexes).

To test if any HMM states expressed higher association with the sleep graphoelements than others, we used *t*-tests comparing each combination of the 19 HMM states ($n_{\text{comparisons}} = [19\times19 - 19] / 2 = 171$). To establish a chance level we compared the original summary measures (correlation, sensitivity, and specificity) to surrogate data created by permuting the HMM state timecourses 1000 times, and re-calculating the summary measures for each permutation. Each permutation consisted in a random switching of the labels of each instance of an HMM state, keeping the number of occurrences of each HMM state and state transition times constant within participants (see Supplementary Figure 19 for an illustration of the permutation principle).

Supplementary Note 5

FC maps of whole-brain network states

The HMM characterised each state by a vector of mean activity and a covariance matrix. These two data statistics may be understood as a dyadic hierarchy of FC information. The mean distribution of a given state gets estimated from the demeaned and standardised timecourses, and hence describes a change away from the grand-average activity level. ROIs that change their activity in the same direction (positive or negative) are thus functionally connected. The covariance matrix of the state then describes the pairwise ROI-to-ROI co-fluctuations within the time periods where the state is active after subtracting the mean activity of the state (i.e. it is the covariance matrix of the residual). Therefore, the state-wise covariance matrices reflect FC within states, above and beyond the largest global FC trends as accounted for by the mean parameter of the Gaussian distributions. The mean activation maps have been overlaid on brain surfaces in Figure 5 and 6 of the main text. The corresponding FC information is represented in Supplementary Figure 5 and 6. The FC matrices were estimated by converting the state-specific covariance matrices to correlations using the Matlab function, corrcov.m. In order to highlight the unique FC characteristics of each HMM state, Supplementary Figure 5 and 6 show differential FC maps. These were computed by taking an FC matrix of a given HMM state and subtracting the average of the FC matrices of the remaining states.

Below we describe the FC information of the whole-brain network states in the order suggested by the transition modules of Figure 4d with an emphasis on how they complement the interpretations of the mean activation maps of the HMM states given in the main text. Supplementary Figures 4 and 5 show the differential FC maps.

For the red module of wakefulness shown in Supplementary Figure 4a, the DMN-like increases in mean activation of HMM state 8 (see Figure 5a) were accompanied by relative increases in FC between occipital and temporal areas, and decreases in FC from the supramarginal gyrus to the posterior cingulate and medial prefrontal areas. Similar decreases in FC were evident for HMM state 2, while increases were particularly clear between the posterior cingulate and medial frontal areas.

For the WASO-related HMM states 5 and 17 the frontal increases in mean activation seen in Figure 5 were complemented by relative frontal increases in FC (see Supplementary Figure 4b).

The whole-brain network states of the white N1-related module are represented in Supplementary Figure 5a. These states generally exhibited opposite polarities in their mean activation between subcortical areas (thalamus and parts of the basal ganglia) and primary sensory

cortical areas (see Figure 6a). In terms of differential FC this subcortico-cortical decoupling was mainly evident for HMM state 1, while increases in connections towards the medial prefrontal cortex and the anterior cingulate was common for all three states.

N2 sleep was dominated by HMM states 3 and 6, and the differential FC maps of these whole-brain network states are shown in Supplementary Figure 5b. Relative increases in FC between superior temporal/inferior parietal areas and medial frontal areas were common, and a few increases in thalamo-cortical connections could be related to association of these HMM states to sleep spindles and the thalamo-cortical mechanism behind the generation of these [73, 74] (although see Supplementary Note 4 for a closer examination of the relationship between the HMM states and sleep graphoelements).

HMM state 16 accounted for the majority of time spent in N3 sleep, and its differential FC is shown in Supplementary Figure 5c. Interestingly, the decreases in mean activation, exhibited broadly throughout the frontal cortices, were complemented by relative increases in frontal and temporo-frontal connectivity. In the same way that the frontal decreases in mean activation could be explained by slow-wave activity (see main text), the relative increases in frontal FC are also in line with the localisation found in EEG-based source modelling of slow waves [75].

To make sure that the differential FC maps were not biased by the effect of the HMM states having different baseline mean activation patterns (as modelled by the mean vector of the Gaussian distribution), we produced equivalent maps using the cosine similarity instead of the covariance matrices outputted by the HMM. Unlike Pearson's correlation, the cosine similarity does not demean the time series, and, therefore, the differences in baselines are accounted for. These maps are shown in Supplementary Figures 17 and 18. This analysis yielded 19 matrices of 90 × 90 cosine similarity values. By taking each cosine similarity matrix and subtracting from it the average of the remaining 18 cosine similarity matrices, we obtained maps equivalent to the differential FC maps, which were based on the covariance information modelled directly by the HMM. As may be seen, when comparing Supplementary Figure 4 to Supplementary Figure 17, and Supplementary Figure 5 to Supplementary Figure 18, the maps are highly similar.

Supplementary Tables

PSG stage	Mean duration *N* = 57 (minutes)	Proportion *N* = 57 (%)	Number of participants out of *N* = 57	Mean duration *N* = 18 (min)	Proportion *N* = 18 (%)	Number of participants out of *N* = 18
**Wake**	24.87 (S.D. 15.39)	47.82	57	12.34 (S.D. 6.61)	23.72	18
**N1**	12.20 (S.D. 9.78)	23.47	57	8.48 (S.D. 2.83)	16.31	18
**N2**	9.71 (S.D. 9.18)	18.68	40	14.66 (S.D. 5.72)	28.19	18
**N3**	5.22 (S.D. 9.02)	10.04	18	16.53 (S.D. 8.39)	31.78	18

Supplementary Table 1. ***Summary details of dataset.*** Left half: presence of each of the four polysomnography (PSG) stages in the full (*N* = 57) dataset. All participants included wakefulness and N1 sleep. Right half: Similar overview for the 18 participants that visited all four PSG stages during their fMRI recordings.

PSG stage	Mean duration *N* = 31 (minutes)	Proportion *N* = 31 (%)	Number of participants out of *N* = 31
**Wake**	8.39 (S.D. 7.94)	16.13	31
**N1**	13.19 (S.D. 7.06)	25.37	31
**N2**	13.54 (S.D. 8.05)	26.05	31
**N3**	4.88 (S.D. 7.92)	9.38	11
**WASO**	12.00 (S.D. 7.68)	23.08	31

Supplementary Table 2. ***Summary details of WASO-subset of the dataset*** In order investigate whether certain HMM states were more likely to occur after consolidated, we extracted the part of the HMM solution that corresponded to the participants, who woke up after having reached N2 sleep. The table shows the distributions of PSG stages (including WASO) across the 31 participants that woke up.

Sleep graphoelement	Mean count (for participants with count > 0)	Number of participants with count > 0 (% of 57)
**Sleep spindles**	29.42 (S.D. 29.36)	33 (57.89 %)
**K-complexes**	32.73 (S.D. 29.19)	37 (64.91 %)

Supplementary Table 3. ***Summary statistics of sleep graphoelements in dataset*** The EEG acquired simultaneously with the fMRI was used to identify sleep graphoelements. This scoring information was re-sampled to the fMRI, such that volumes during which either a sleep spindle or a K-complex occurred were marked. The first column of the table shows the mean number of occurrences of each sleep graphoelement after this re-sampling. The mean value is calculated within the participants that included at least one of the given graphoelement. The number of participants including at least on sleep spindle or K-complex is shown in the first and second row, respectively, of the second column of the table.

Supplementary Figures

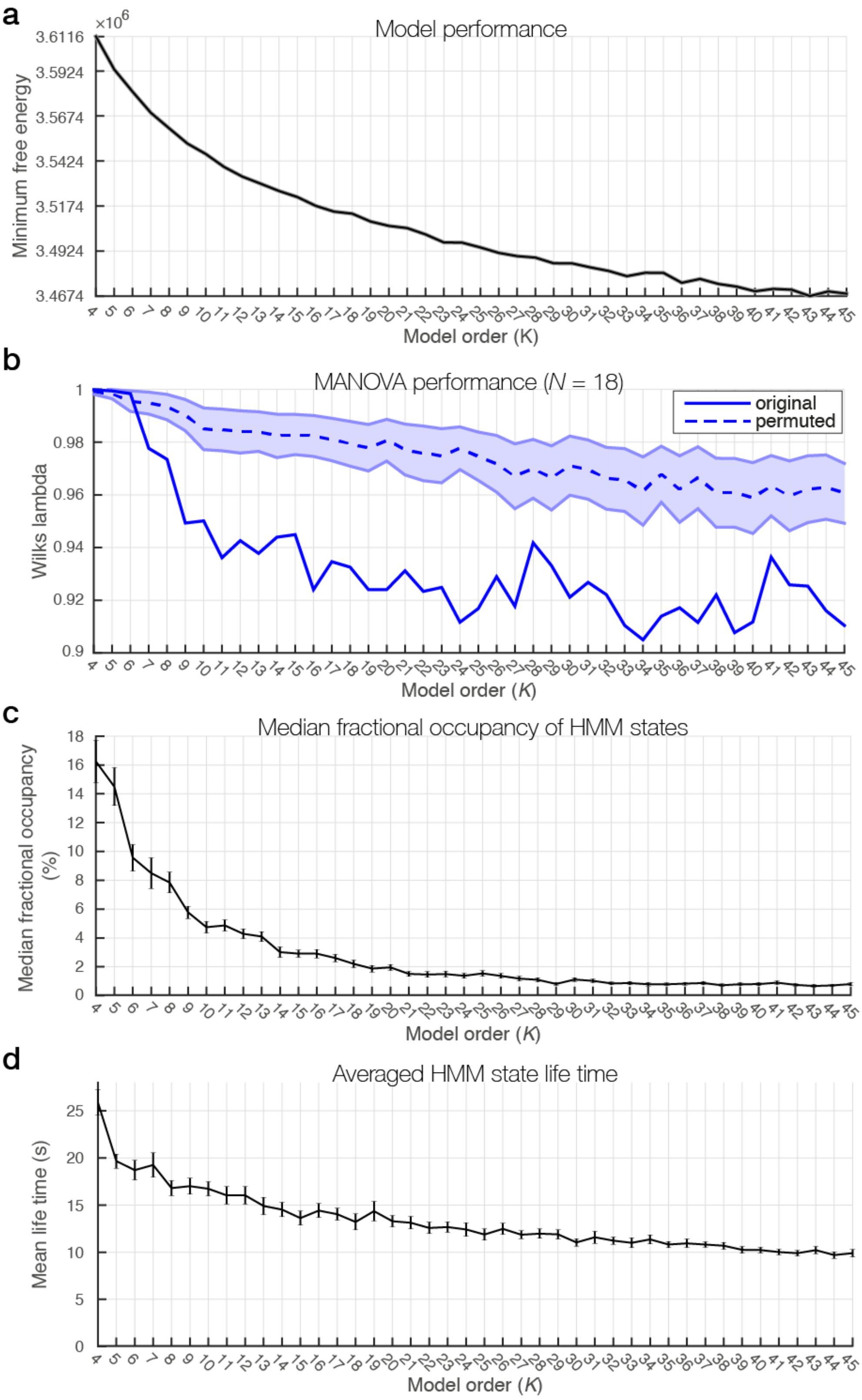

Supplementary Figure 1. ***Summary measures of HMM solutions across a range of model orders.*** **a** The minimum free-energy on the VB inference decreased monotonically for increasing model orders. **b** Wilk's lambda is an output of the MATLAB function manova1, and describes how well the HMM state timecourses

could be grouped according to the EEG-based sleep scoring. Lower values correspond to a better fit. Here Wilk's lambda is shown for the part of HMM solutions corresponding to the 18 participants that included all four PSG stages and across a range of model orders. The solid blue line depicts the output of the MANOVA run with the original sleep scoring, while the dashed blue line corresponds to the average Wilk's lambda from 1000 MANOVAs where the sleep scoring was randomly permuted. Error zones correspond to the standard deviations of the permuted cases. Note how the original sleep scoring expressed significantly better fitting with the HMM state timecourses for model orders above $K = 7$, and how Wilk's lambda appeared to stagnate somewhere between $10 < K > 20$. **c** The development of the median fractional occupancy as a function of HMM model order. Vertical error bars indicate the standard errors across participants ($N = 18$). Note how the median fractional occupancy stagnates from around $K \sim 19$, indicating that the addition of more states did not split existing (at lower model orders) states, but resulted in the addition of 'sporadic' states. **d** HMM state life time averaged across states and participants ($N = 18$) as a function of model order. Error bars indicate the standard errors across participants. Note how the curve stagnates around $K \sim 18$.

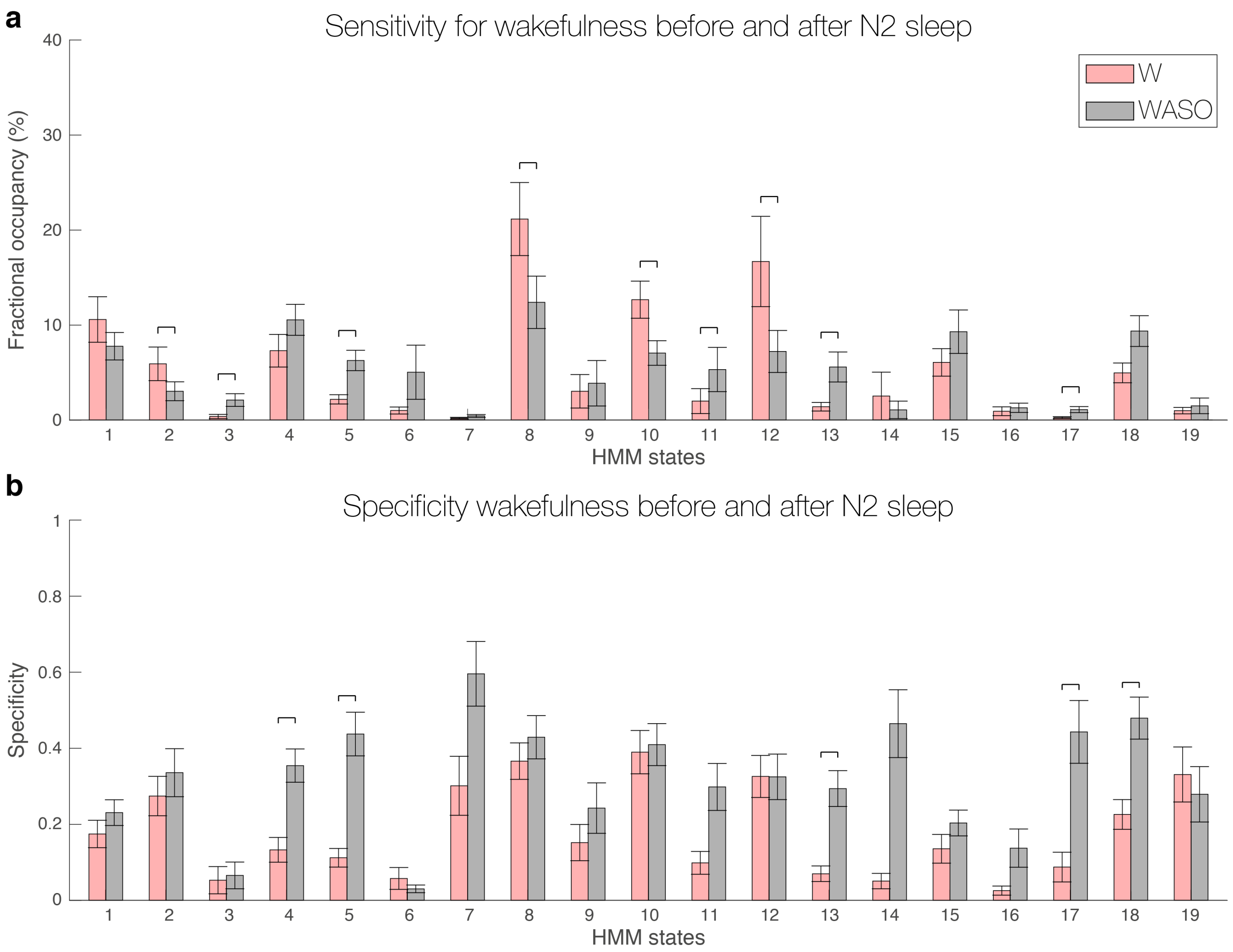

Supplementary Figure 2. ***Sensitivity and specificity of HMM states when separating wakefulness before and after sleep*** (supplement to Figure 3).a and b corresponds to a and b of Figure 3 respectively, but for the 31 participants that woke up after having reached N2 sleep. Wake after sleep onset (WASO) was defined as polysomnographically estimated wakefulness following N2 sleep, and is represented by the black bars, while wakefulness (W) was defined as periods of PSG-estimated wakefulness prior to N2 sleep. The coloured bars and error bars show the average and standard error, respectively, across participants. To highlight the differences between W and WASO, we have excluded the remaining sleep stages from this plot. Horizontal lines show significant differences with p-values < 0.01, as evaluated with paired t-tests and permutation testing. Note how HMM states forming the black module in the transition map of Figure 4, showed higher specificity for WASO compared to W.

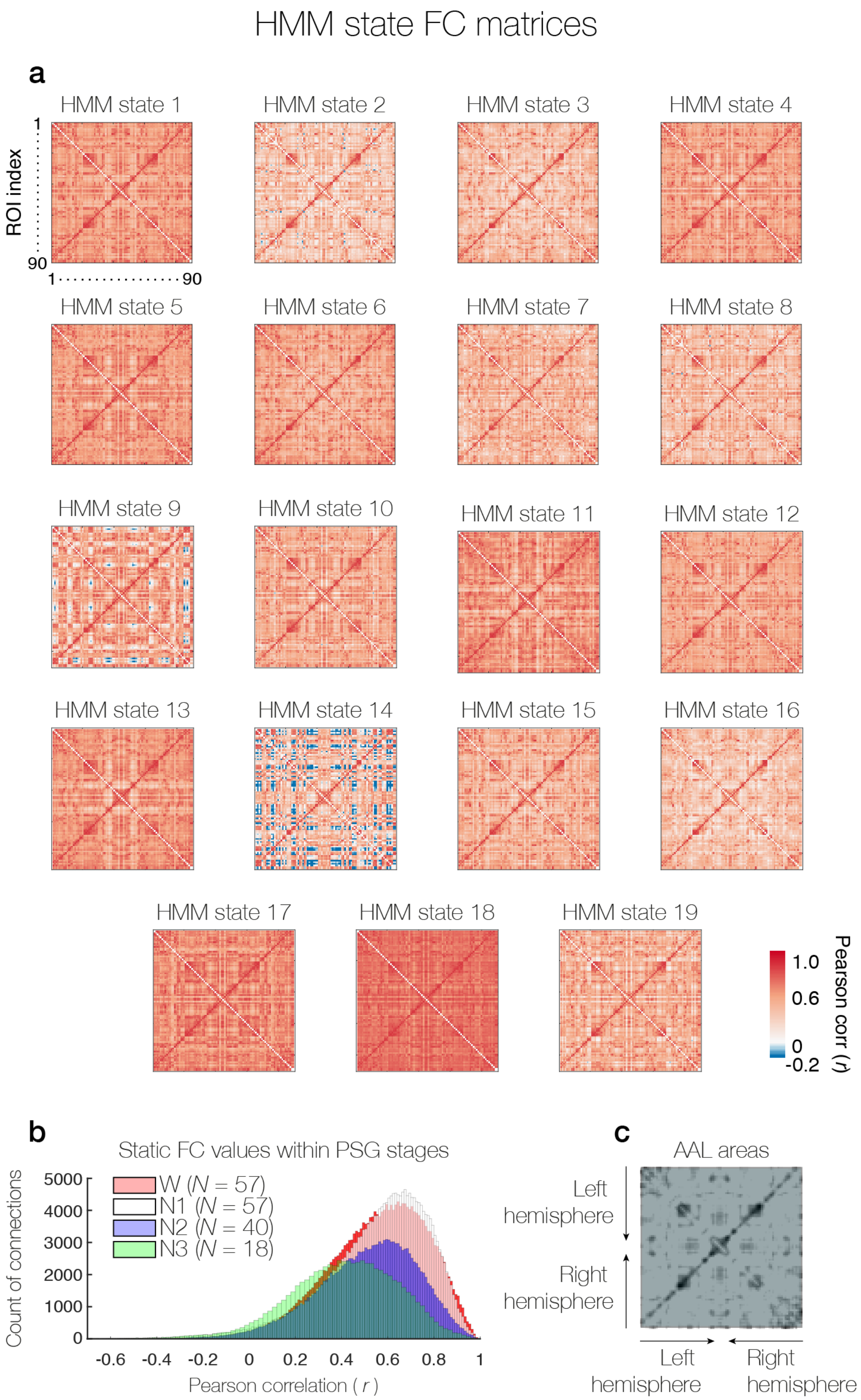

Supplementary Figure 3. ***HMM state FC matrices.*** **a** The 90 × 90 FC matrices for the 19 HMM states were calculated as conversion from the state covariance matrices output directly by the HMM. Covariance values were converted to Pearson's correlation values. Each entry of a matrix corresponds to the pairwise FC between two AAL ROIs. **b** The FC matrices of the HMM states were skewed towards positive correlations. To show that this was a feature of the ROI timecourses and not an effect of the HMM, we have included

histograms of the super-diagonal elements of the static FC matrices computed directly from the fMRI data within the PSG stages. **c** The ROIs of the FC matrices in **a** have been reordered such that the first and third quadrants include cross-hemispheric connections, while inter-hemispheric connections are shown in the second and fourth quadrants.

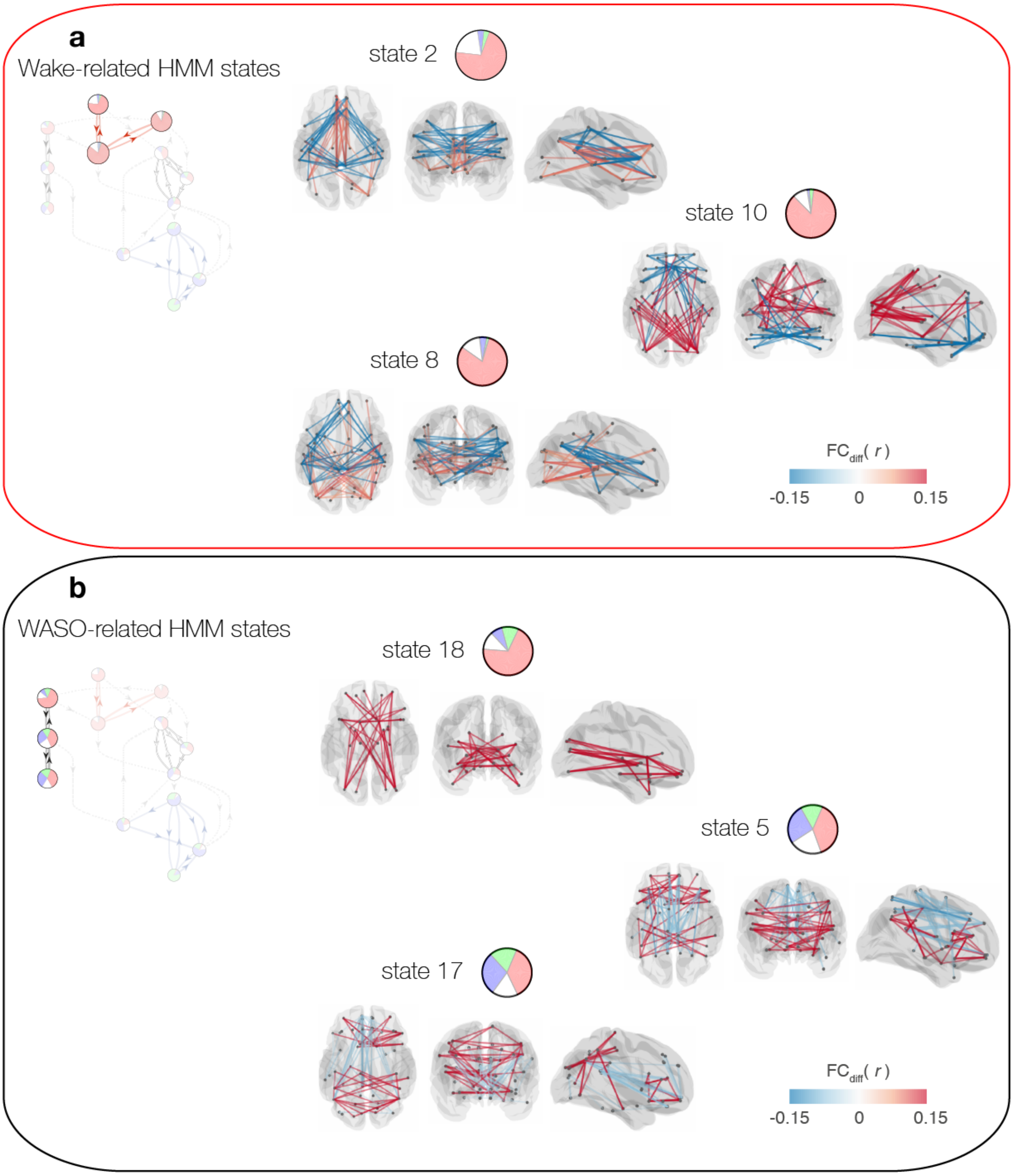

Supplementary Figure 4. ***Differential FC maps of wakefulness-related HMM states*** (supplement to Figure 5). Differential FC maps were computed by taking an FC matrix of a given HMM state and subtracting the average of the FC matrices of the remaining states. Maps show the 1% most negative and 1% most positive weights of each state **a** Differential FC maps of wakefulness prior to sleep. Note the relative increases in connections between occipital and temporal areas, as well as the decreases between supramarginal gyrus and the posterior cingulate cortex. **b** Differential FC maps of wakefulness after sleep onset (WASO). These states were in general characterised by relative increases in frontal connections.

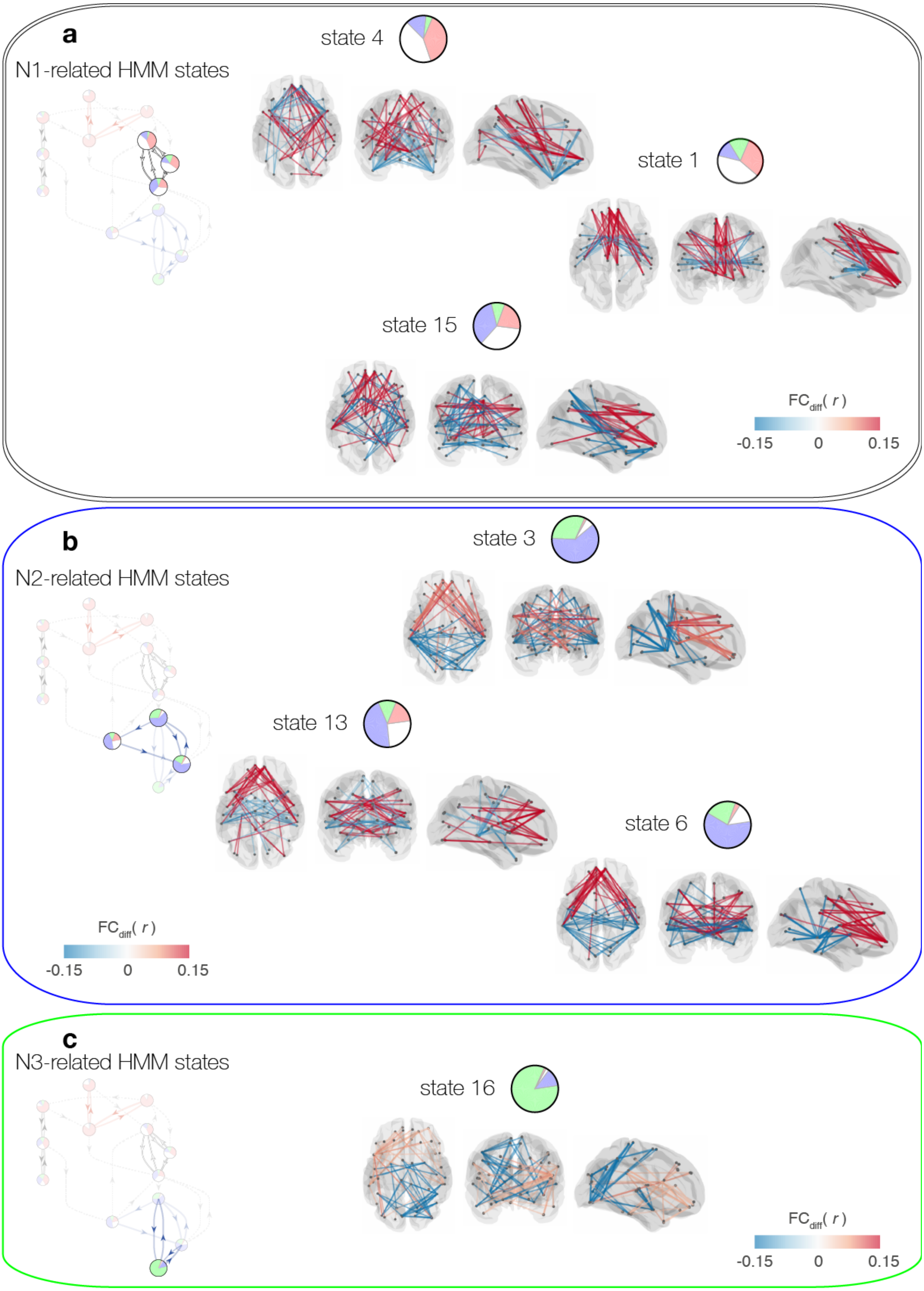

Supplementary Figure 5. ***Differential FC maps of sleep-related HMM states*** (supplement to Figure 6). **a** Differential FC maps of HMM states related to N1 sleep. Note the decreased FC between subcortical and

temporal/parietal areas of HMM state 1. **b** Differential FC maps of N2-related HMM states. A common trait of these three states was increased FC in temporal/inferior parietal areas and medial frontal. **c** N3-related differential FC maps. Note the relative increases in frontal FC. Maps show the 1% most negative and 1% most positive weights of each state

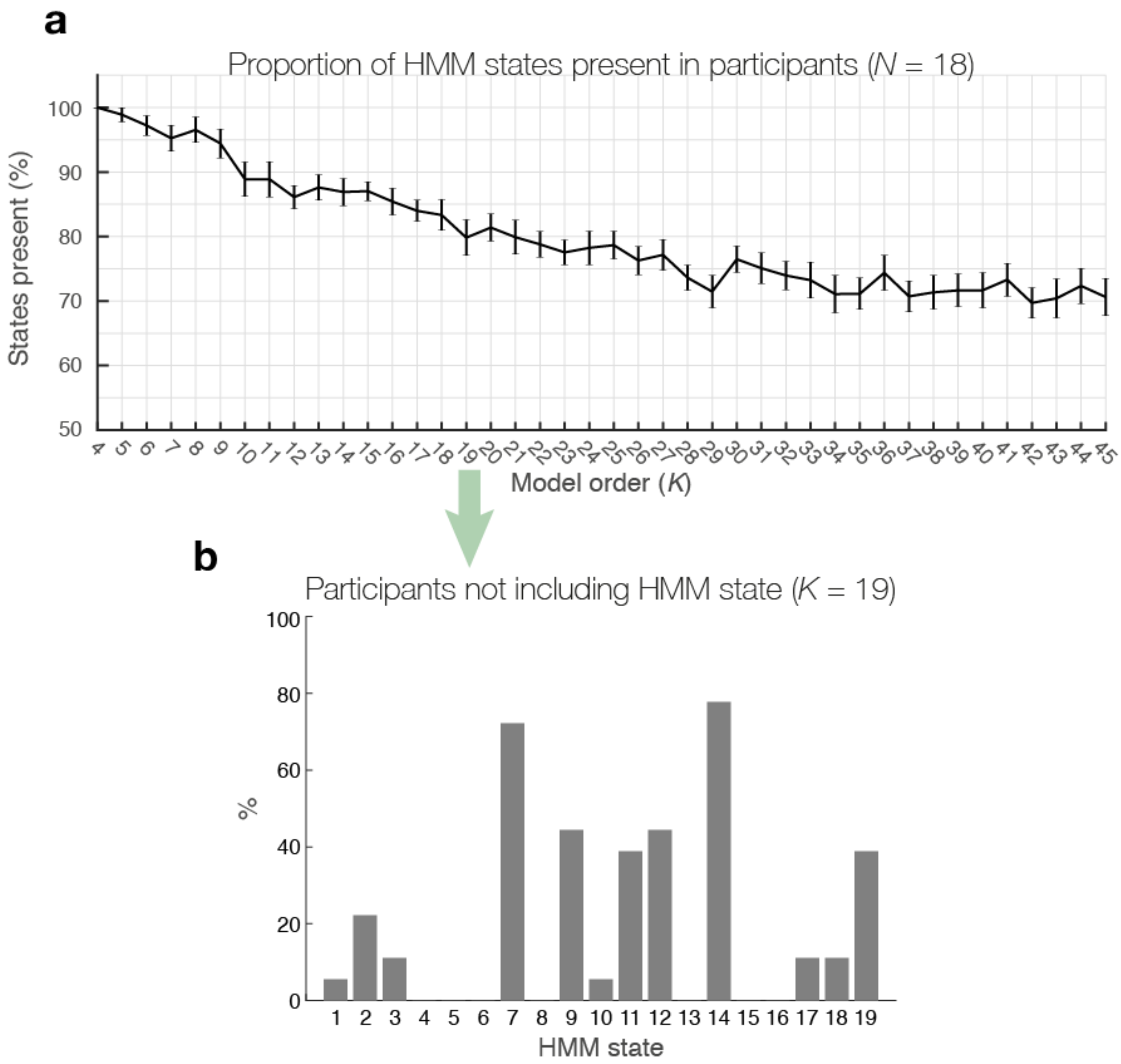

Supplementary Figure 6. ***Summary of inconsistent HMM states across participants.*** **a** The graph shows the proportion of HMM states that were present in all participants as a function of model order. Included here are the 18 participants that reached all four PSG stages during their recording session. As the model order increased the probability of finding all HMM states in all participants dropped. Error bars show the standard error across participants. **b** The solution presented in the main text (K = 19) is summarised in terms of the presence of the individual HMM states. The bars show the proportion of participant in which the HMM states were not present. As evident, six HMM states (7, 9, 11, 12, 14, and 19) were present in less than 60 % of the participants. We call these 'sporadic' HMM states, as they did not model data traits consistent across participants.

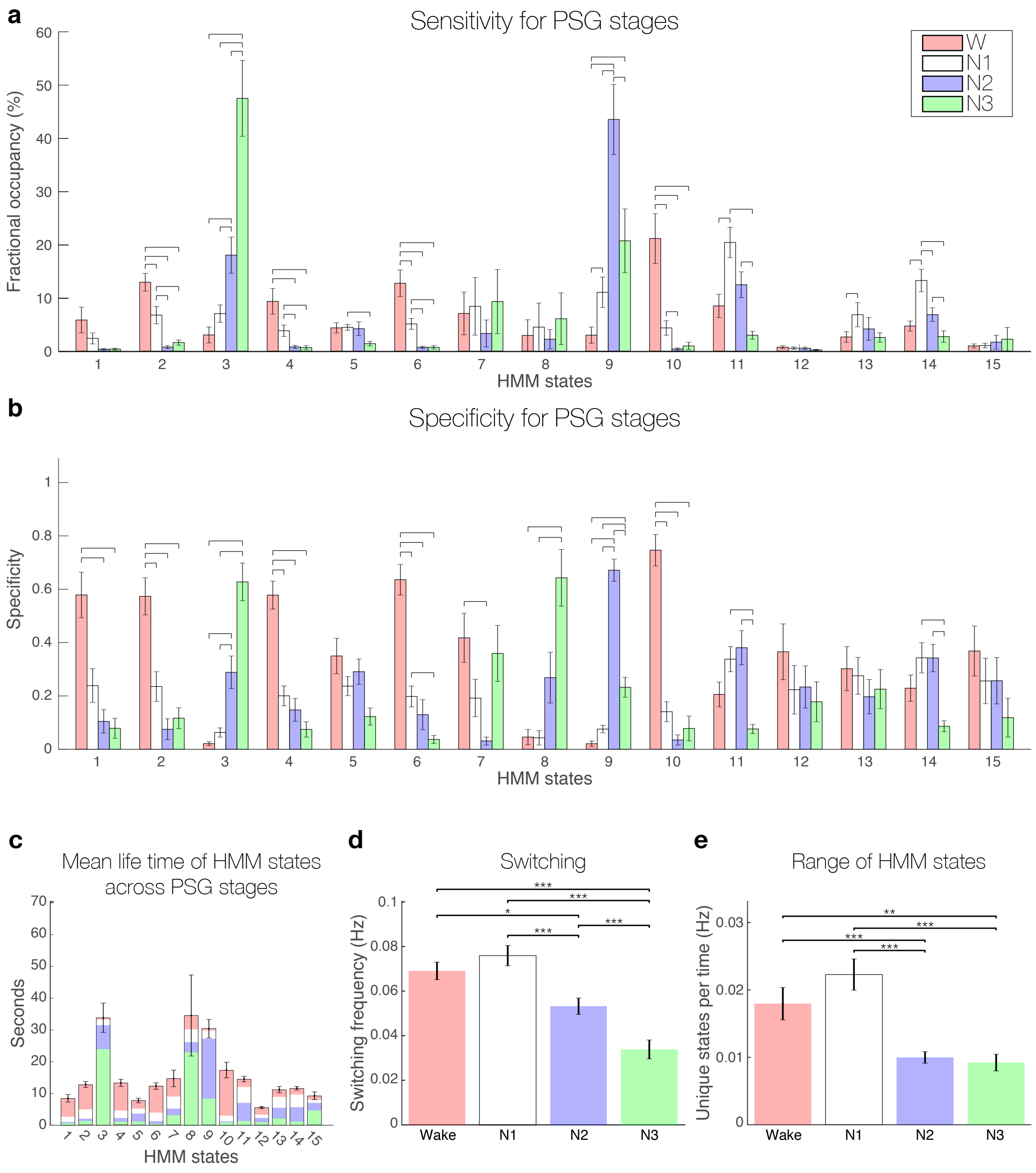

Supplementary Figure 7. ***K = 15, Sensitivity and specificity of HMM states and dynamics within polysomnography stages*** (supplement to Figure 3). **a** Fractional occupancies of each of the 15 HMM states computed within the four PSG stages corresponded to the PSG-sensitivity of the whole-brain network states. The coloured bars and error bars show the average and standard error, respectively, across the 18 participants that included all four PSG stages. **b** PSG-specificity of the HMM states for each of the four PSG stages. Specificity corresponds to the probability of an HMM state occurring within a PSG stage. The bars represent the group average and the error bars the standard error ($N = 18$). In **a** and **b** horizontal lines show significant differences within HMM states, with p-values < 0.01 as evaluated through paired t-tests and permutation testing. **c** The mean life times of the 15 HMM states are shown by the bars, representing values averaged across the 18 participants. Error-bars represent the standard error across participants. Each HMM state is coloured according to the probability of finding it within each of the four PSG stages, i.e. their PSG

specificity. **d** The dynamics of the HMM transitions were calculated within each of the four PSG stages, in terms of switching frequency (‘Switching’), and **e** the number of different HMM states visited per time (‘Range of HMM states’). In **d** and **e** error bars represent standard error across participants and significant differences between PSG stages are denoted by stars: * $p < 0.05$, ** $p < 0.01$, *** $p < 0.001$.

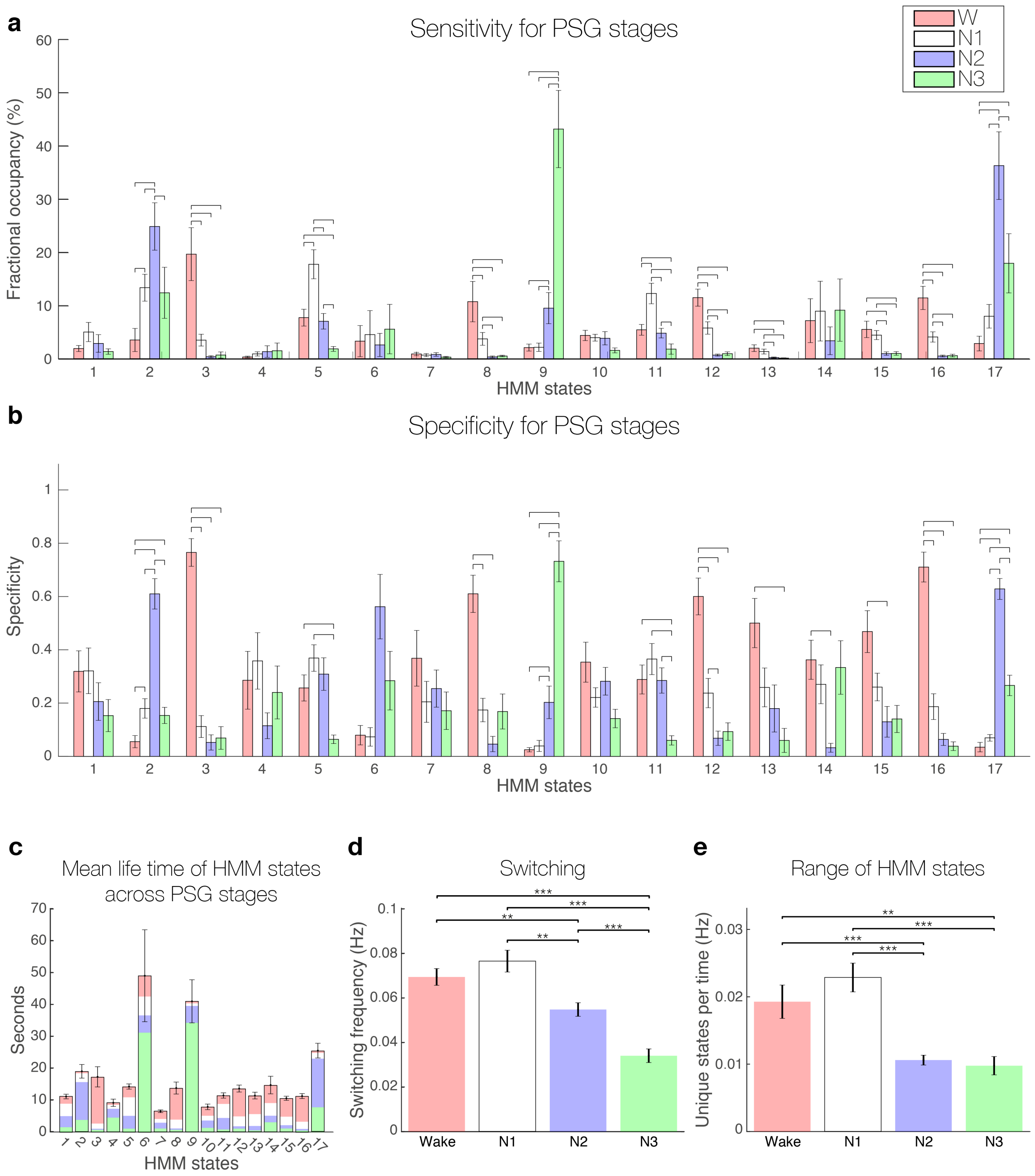

Supplementary Figure 8. ***K = 17, Sensitivity and specificity of HMM states and dynamics within polysomnography stages*** (supplement to Figure 3). **a** Fractional occupancies of each of the 17 HMM states computed within the four PSG stages corresponded to the PSG-sensitivity of the whole-brain network states. The coloured bars and error bars show the average and standard error, respectively, across the 18 participants that included all four PSG stages. **b** PSG-specificity of the HMM states for each of the four PSG stages. Specificity corresponds to the probability of an HMM state occurring within a PSG stage. The bars represent the group average and the error bars the standard error ($N = 18$). In **a** and **b** horizontal lines show significant differences within HMM states, with p-values < 0.01 as evaluated through paired t-tests and permutation testing. **c** The mean life times of the 17 HMM states are shown by the bars, representing values averaged across the 18 participants. Error-bars represent the standard error across participants. Each HMM state is coloured according to the probability of finding it within each of the four PSG stages, i.e. their PSG

specificity. **d** The dynamics of the HMM transitions were calculated within each of the four PSG stages, in terms of switching frequency ('Switching'), and **e** the number of different HMM states visited per time ('Range of HMM states'). In **d** and **e** error bars represent standard error across participants and significant differences between PSG stages are denoted by stars: * $p < 0.05$, ** $p < 0.01$, *** $p < 0.001$.

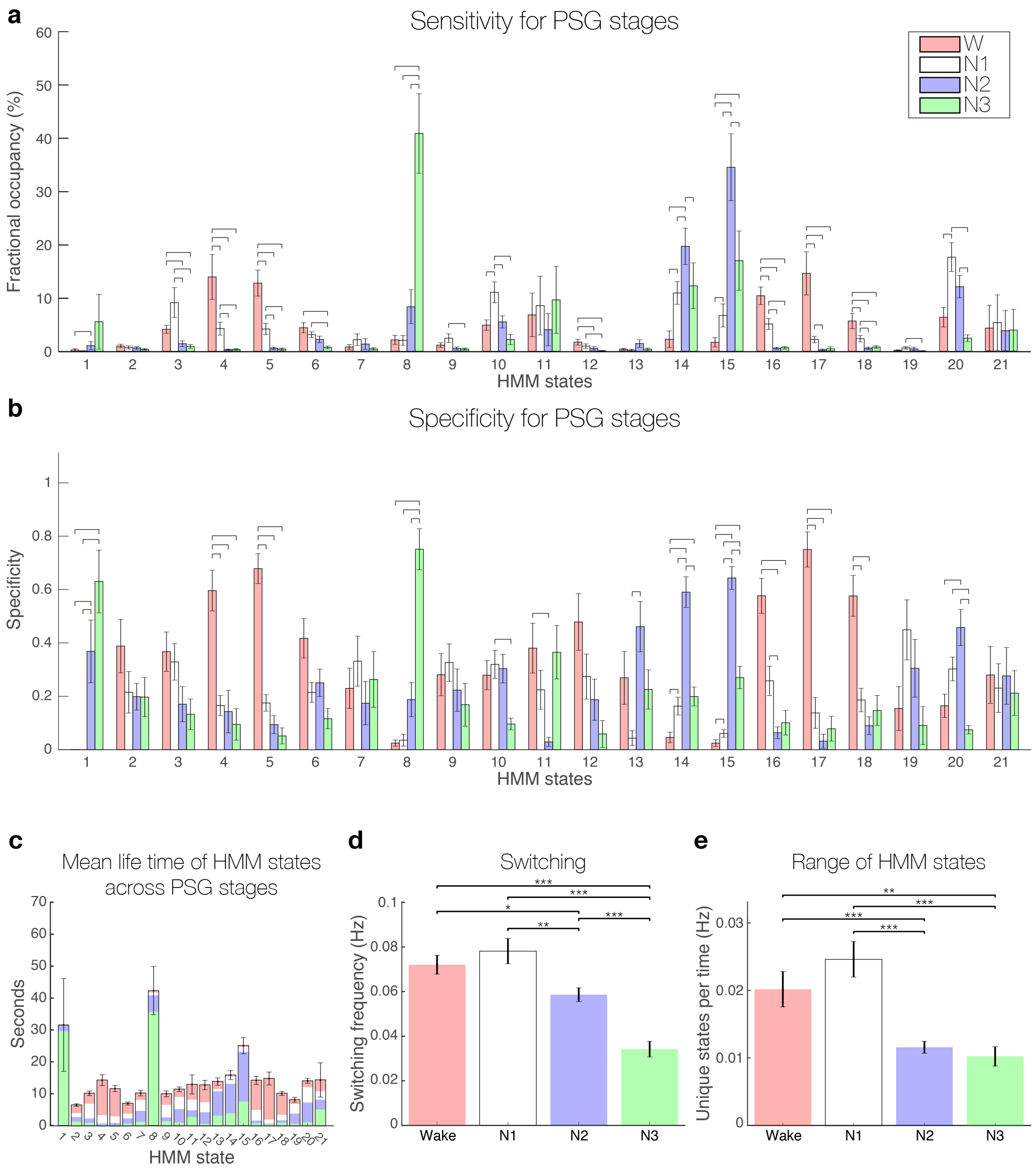

Supplementary Figure 9. ***K = 21, Sensitivity and specificity of HMM states and dynamics within polysomnography stages*** (supplement to Figure 3). **a** Fractional occupancies of each of the 21 HMM states computed within the four PSG stages corresponded to the PSG-sensitivity of the whole-brain network states. The coloured bars and error bars show the average and standard error, respectively, across the 18 participants that included all four PSG stages. **b** PSG-specificity of the HMM states for each of the four PSG stages. Specificity corresponds to the probability of an HMM state occurring within a PSG stage. The bars represent the group average and the error bars the standard error ($N = 18$). In **a** and **b** horizontal lines show significant differences within HMM states, with p-values < 0.01 as evaluated through paired t-tests and permutation testing. **c** The mean life times of the 21 HMM states are shown by the bars, representing values averaged across the 18 participants. Error-bars represent the standard error across participants. Each HMM state is coloured according to the probability of finding it within each of the four PSG stages, i.e. their PSG

specificity. **d** The dynamics of the HMM transitions were calculated within each of the four PSG stages, in terms of switching frequency ('Switching'), and **e** the number of different HMM states visited per time ('Range of HMM states'). In **d** and **e** error bars represent standard error across participants and significant differences between PSG stages are denoted by stars: * $p < 0.05$, ** $p < 0.01$, *** $p < 0.001$.

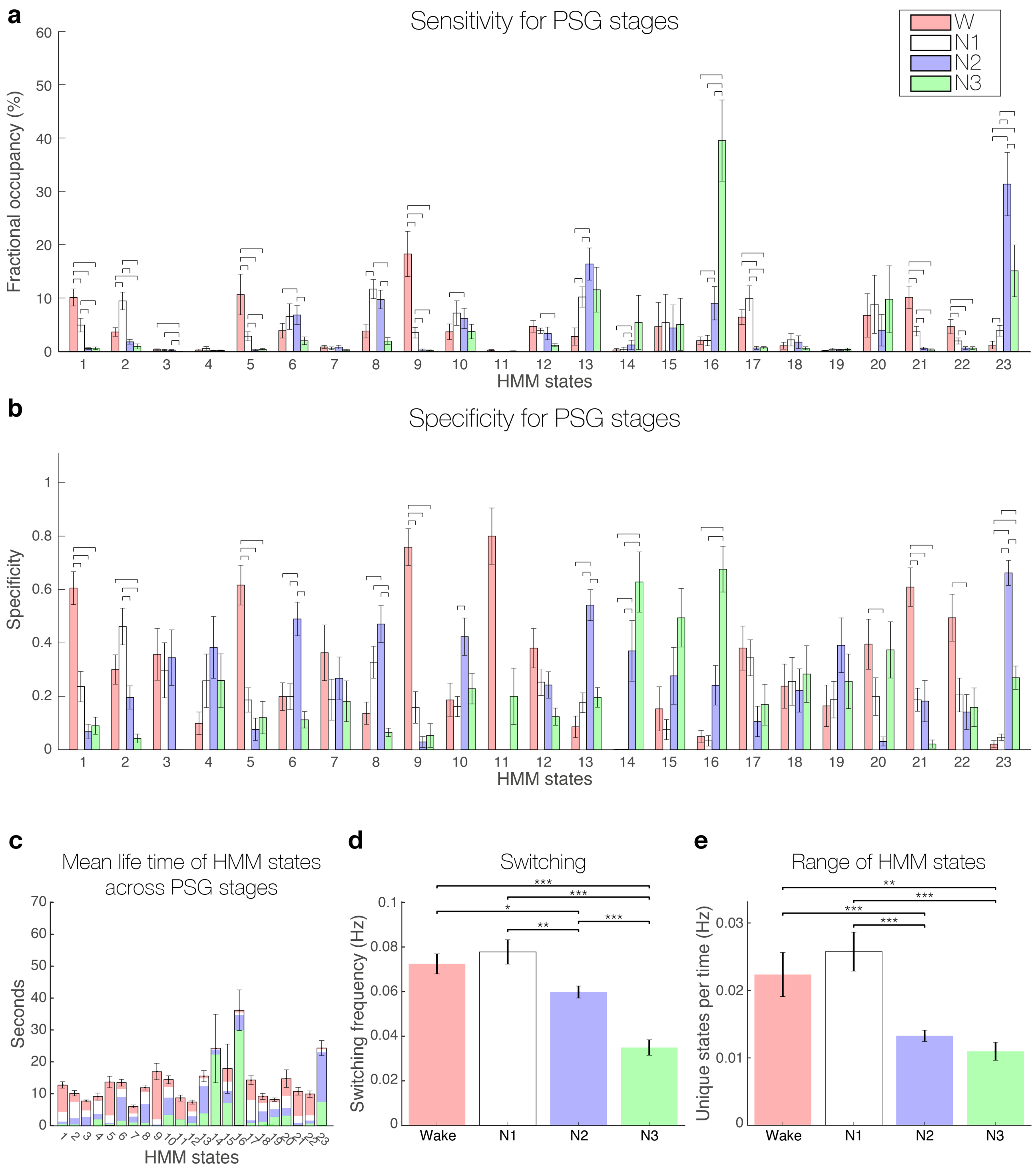

Supplementary Figure 10. ***K = 23, Sensitivity and specificity of HMM states and dynamics within polysomnography stages*** (supplement to Figure 3). **a** Fractional occupancies of each of the 23 HMM states computed within the four PSG stages corresponded to the PSG-sensitivity of the whole-brain network states. The coloured bars and error bars show the average and standard error, respectively, across the 18 participants that included all four PSG stages. **b** PSG-specificity of the HMM states for each of the four PSG stages. Specificity corresponds to the probability of an HMM state occurring within a PSG stage. The bars represent the group average and the error bars the standard error ($N = 18$). In **a** and **b** horizontal lines show significant differences within HMM states, with p-values < 0.01 as evaluated through paired t-tests and permutation testing. **c** The mean life times of the 23 HMM states are shown by the bars, representing values averaged across the 18 participants. Error-bars represent the standard error across participants. Each HMM state is coloured according to the probability of finding it within each of the four PSG stages, i.e. their PSG

specificity. **d** The dynamics of the HMM transitions were calculated within each of the four PSG stages, in terms of switching frequency ('Switching'), and **e** the number of different HMM states visited per time ('Range of HMM states'). In **d** and **e** error bars represent standard error across participants and significant differences between PSG stages are denoted by stars: * $p < 0.05$, ** $p < 0.01$, *** $p < 0.001$.

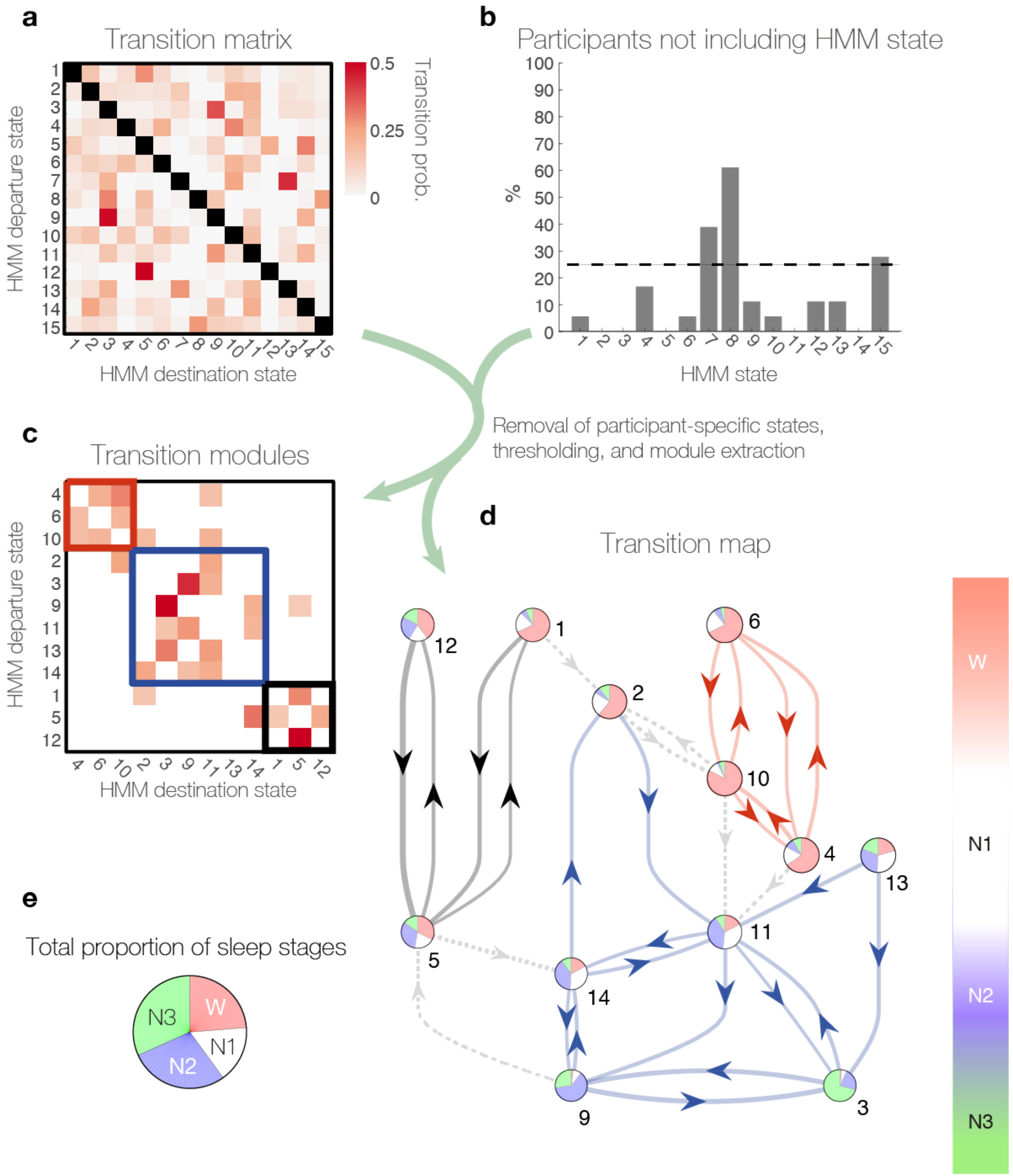

Supplementary Figure 11. ***K = 15 Investigating transitions between whole-brain network states.*** (supplement to Figure 4). **a** The figure shows the 15 × 15 transition probability matrix of the HMM states calculated for the 18 participants that included all four PSG stages in their respective scanning session. This quantifies the likelihood of transitioning from any given state to any other state, giving each matrix entry: probability of (departure state, destination state). **b** A few HMM states were 'sporadic' and did not occur consistently across participants. HMM states not occurring in more than 25% of the participants were excluded. **c** The strongest transitions of the consistent HMM states were partitioned through a modularity analysis, and reorganised in a matrix according to the four resulting modules. **d** The transitions shown in **c** are presented as a transition map with each state depicted as a pie plot expressing its specificity for each of the four PSG stages. Arrows show the direction of the transitions with thickness proportional to the transition

probability. Note the similar overall structure of the transition map to the one presented in Figure 4 of the main text. Unlike the other numbers of states tested, K = 15 only yielded 3 modules, suggesting that the white and the blue module of Figure 4 have merged together in one. **e** Pie chart showing the total proportion PSG stages within the 18 participants.

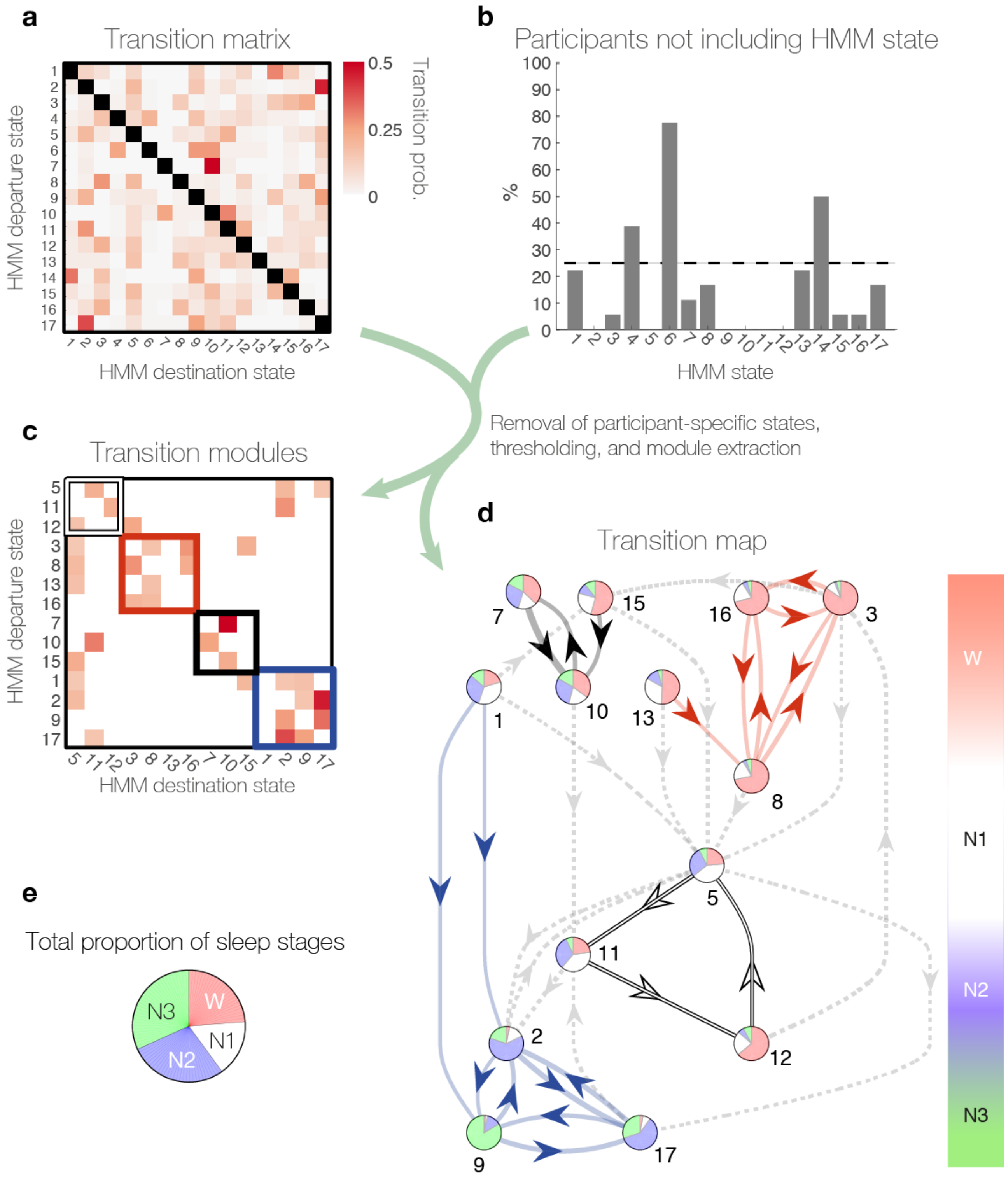

Supplementary Figure 12. ***K = 17 Investigating transitions between whole-brain network states.*** (supplement to Figure 4). **a** The figure shows the 17 × 17 transition probability matrix of the HMM states calculated for the 18 participants that included all four PSG stages in their respective scanning session. This quantifies the likelihood of transitioning from any given state to any other state, giving each matrix entry: probability of (departure state, destination state). **b** A few HMM states were 'sporadic' and did not occur consistently across participants. HMM states not occurring in more than 25% of the participants were excluded. **c** The strongest transitions of the consistent HMM states were partitioned through a modularity analysis, and reorganised in a matrix according to the four resulting modules. **d** The transitions shown in **c** are presented as a transition map with each state depicted as a pie plot expressing its specificity for each of

the four PSG stages. Arrows show the direction of the transitions with thickness proportional to the transition probability. Note the similar overall structure of the transition map to the one presented in Figure 4 of the main text, including 4 separated transition modules. **e** Pie chart showing the total proportion PSG stages within the 18 participants.

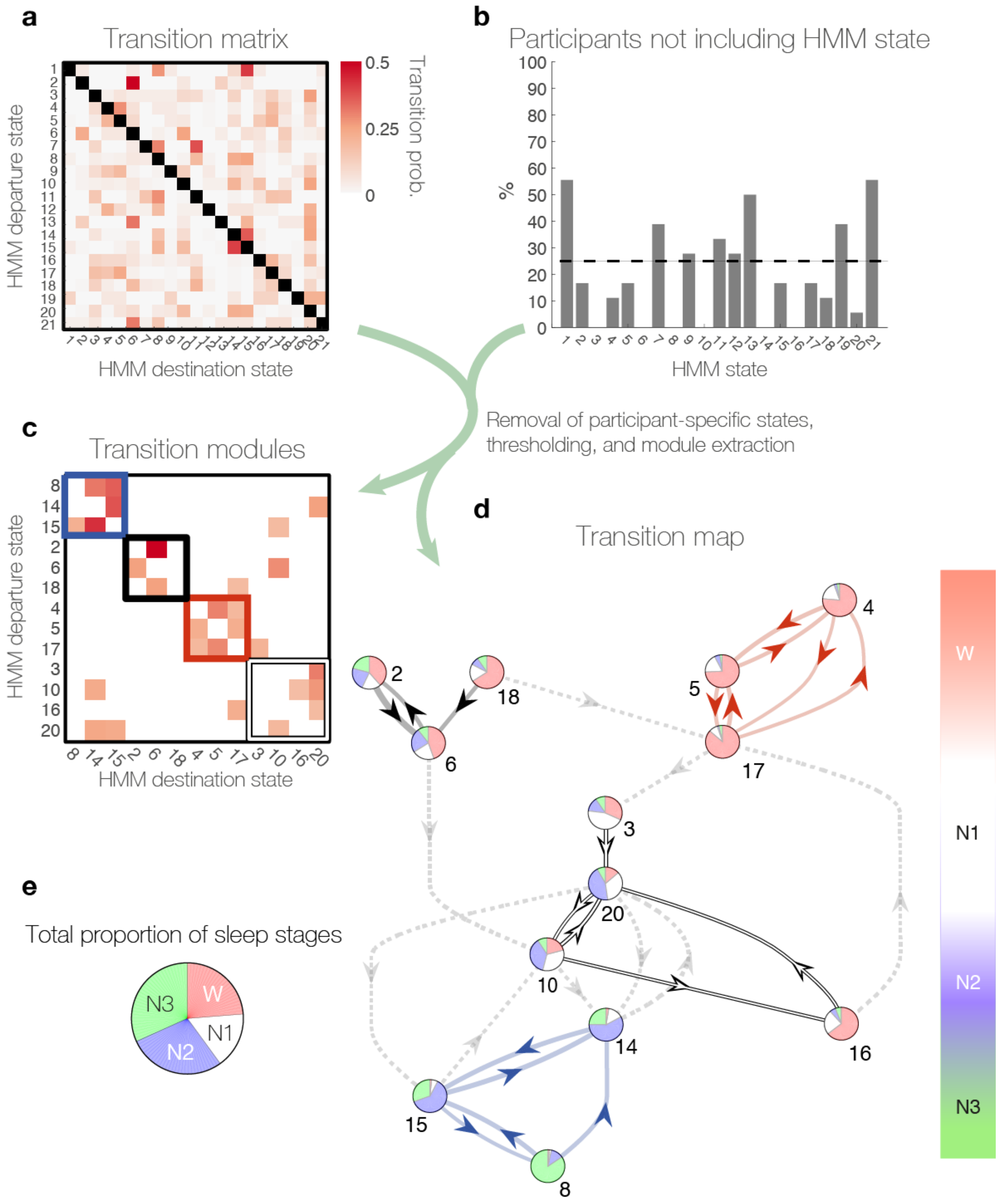

Supplementary Figure 13. ***K = 21 Investigating transitions between whole-brain network states.*** (supplement to Figure 4). **a** The figure shows the 21 × 21 transition probability matrix of the HMM states calculated for the 18 participants that included all four PSG stages in their respective scanning session. This quantifies the likelihood of transitioning from any given state to any other state, giving each matrix entry: probability of (departure state, destination state). **b** A few HMM states were 'sporadic' and did not occur consistently across participants. HMM states not occurring in more than 25% of the participants were excluded. **c** The strongest transitions of the consistent HMM states were partitioned through a modularity

analysis, and reorganised in a matrix according to the four resulting modules. **d** The transitions shown in **c** are presented as a transition map with each state depicted as a pie plot expressing its specificity for each of the four PSG stages. Arrows show the direction of the transitions with thickness proportional to the transition probability. Note the similar overall structure of the transition map to the one presented in Figure 4 of the main text, including 4 separated transition modules. **e** Pie chart showing the total proportion PSG stages within the 18 participants.

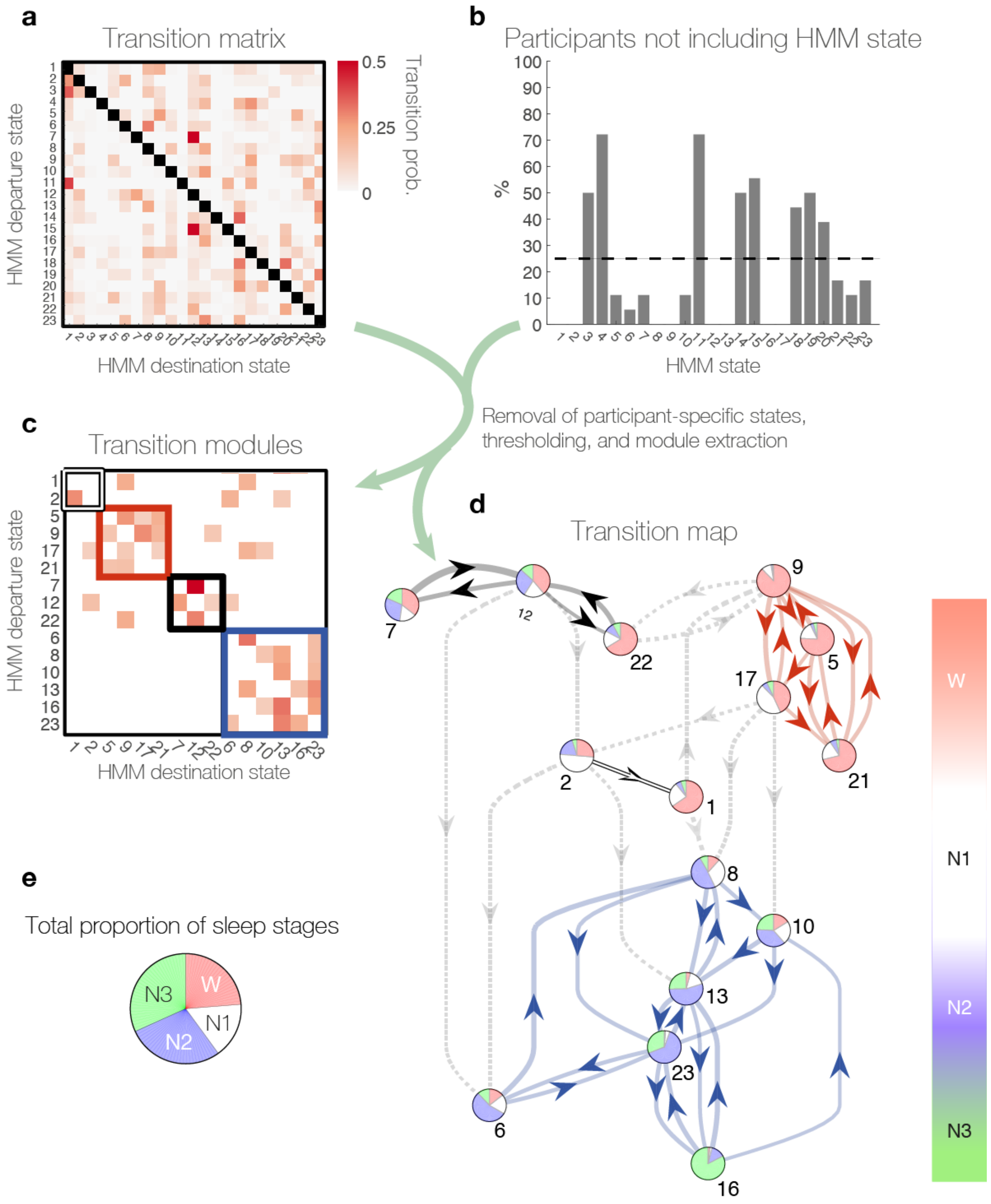

Supplementary Figure 14. ***K = 23 Investigating transitions between whole-brain network states.*** (supplement to Figure 4). **a** The figure shows the 23 × 23 transition probability matrix of the HMM states calculated for the 18 participants that included all four PSG stages in their respective scanning session. This quantifies the likelihood of transitioning from any given state to any other state, giving each matrix entry: probability of (departure state, destination state). **b** A few HMM states were 'sporadic' and did not occur consistently across participants. HMM states not occurring in more than 25% of the participants were

excluded. **c** The strongest transitions of the consistent HMM states were partitioned through a modularity analysis, and reorganised in a matrix according to the four resulting modules. **d** The transitions shown in **c** are presented as a transition map with each state depicted as a pie plot expressing its specificity for each of the four PSG stages. Arrows show the direction of the transitions with thickness proportional to the transition probability. Note the similar overall structure of the transition map to the one presented in Figure 4 of the main text, including 4 separated transition modules. **e** Pie chart showing the total proportion PSG stages within the 18 participants.

a Distances between state distributions across different HMM initialisations

b Temporal robustness across different HMM initialisation

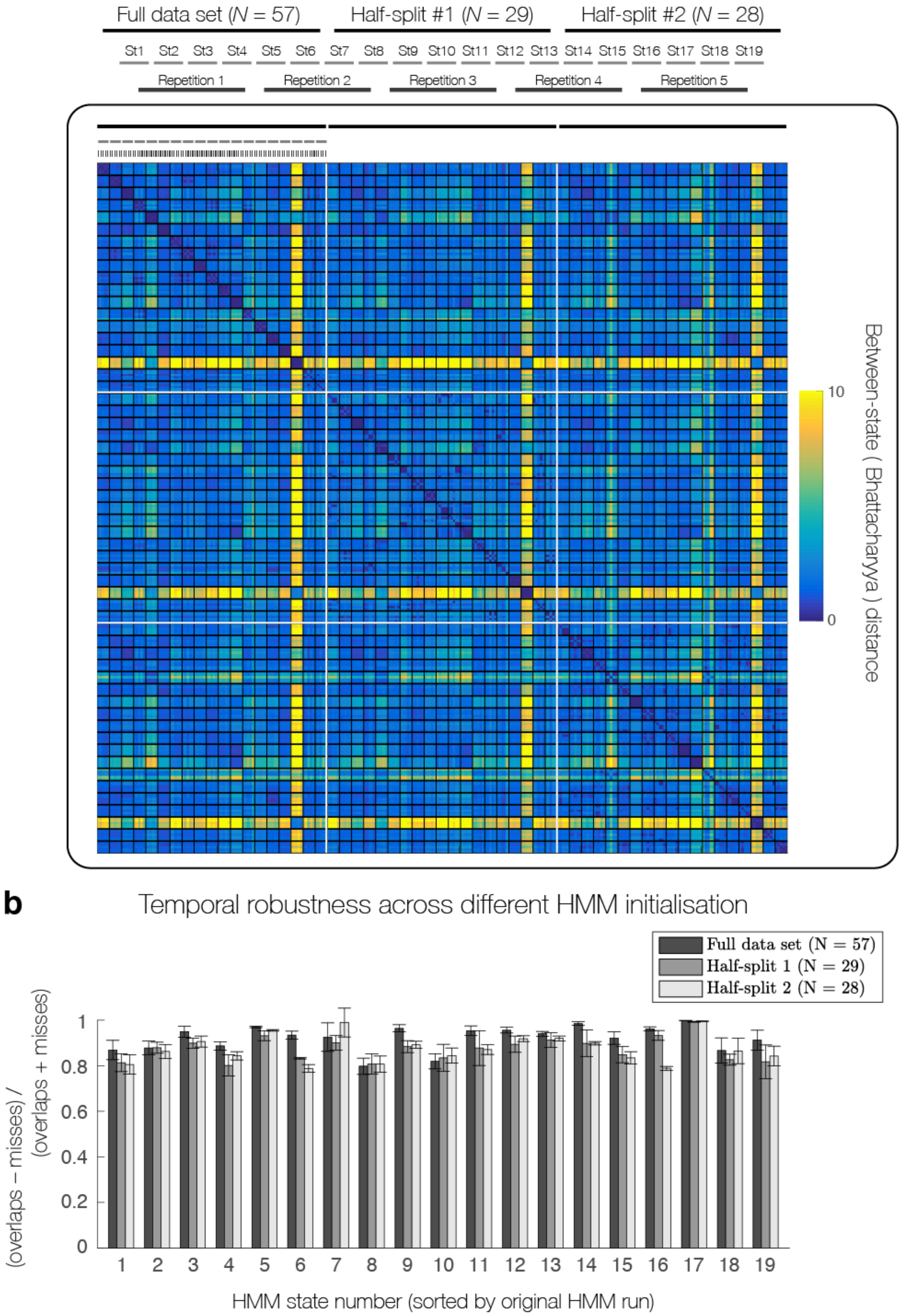

Supplementary Figure 15. ***Robustness across different random initialisation of the HMM.*** **a** To make sure that the states inferred by the HMM were not contingent on the initialisation, we ran the HMM with 19 states an additional four times on the full dataset ('Full data set', *N* = 57), and five times on each of the two half-

splits of the data (‘Half-split 1’, $N = 29$, and ‘Half-split 2’ $N = 28$). The 19 HMM states from a given repetition were matched to the HMM states of the original solution, using the Munkres algorithm, based on the intra-solution state-distances (measured using the Bhattacharyya distance between the states’ Gaussian distributions). The matrix shows the pairwise Bhattacharyya distances between the Gaussian distributions following the matching of the HMM states from the different repetition runs. As indicated by the labelling above the matrix, the white borders demarcate states from the various data splits. The smaller black squares surround 5 repetitions of each of the 19 HMM states. The consistent appearance within each of the 9 white squares is a sign that the HMM inferred states with consistent Gaussian distributions. **b** For a pair of HMM states (one original and one from a repetition run) the temporal correspondence was quantified as the ratio between time points of overlap (simultaneous activity or inactivity) and time points of misses. The bar plot shows mean values and error bars show the standard deviations within data-splits. Note how that temporal overlaps outweighed misses for all runs of the HMM.

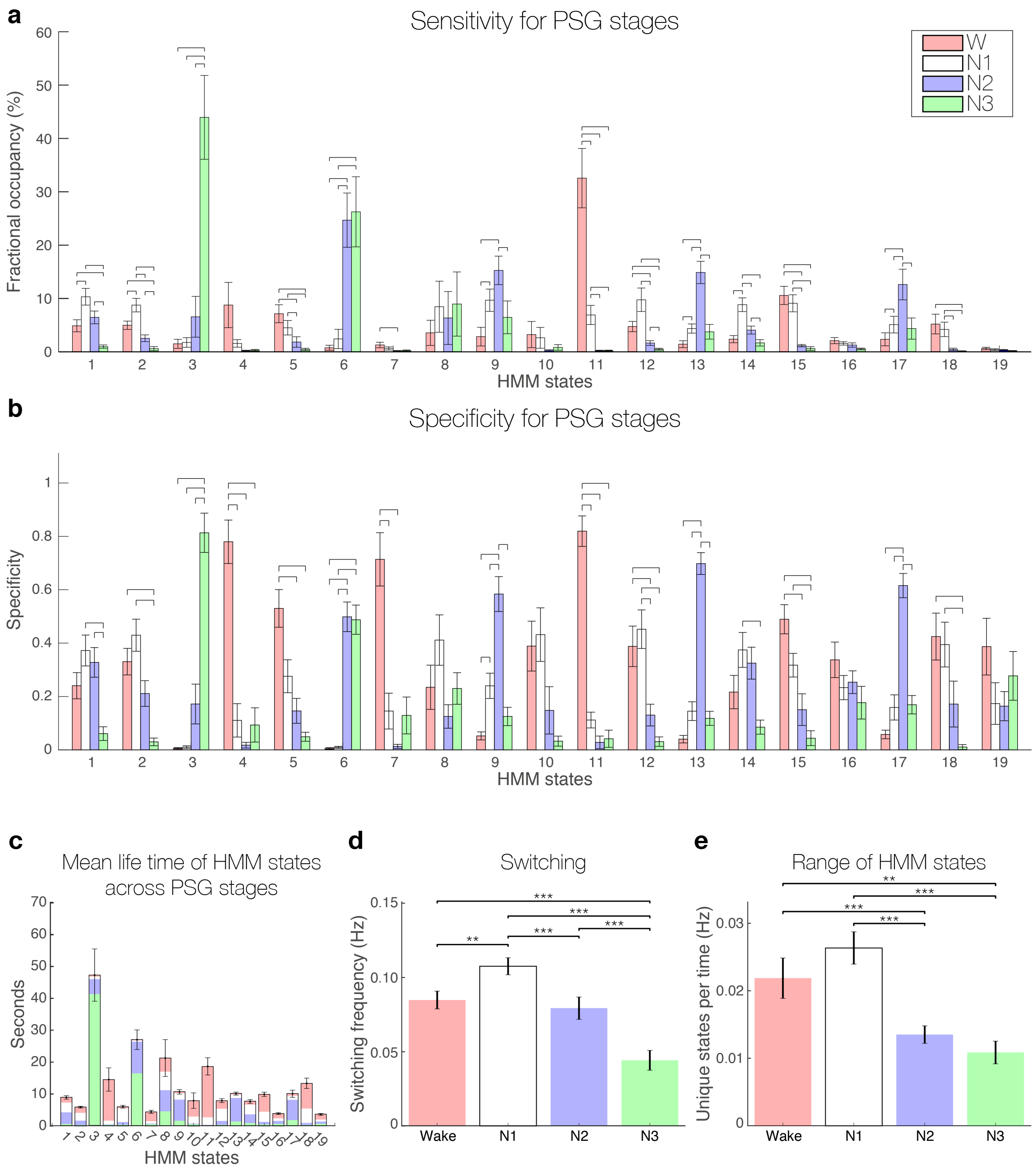

Supplementary Figure 16. ***Robustness of HMM results without the use of temporal filter*** (supplement to Figure 3). All plots were computed in the same way as for Figure 3, using an HMM with 19 states. The only difference was that the BOLD data was not temporally filtered. Note in **a** and **b** how, similarly to the results of the main text, select HMM states were sensitive and specific for certain PSG stages (although not for N1 sleep). In **c** the overall mean life time of the HMM states is decreased compared to the results using a low-pass temporal filter (compare with Figure 3). As a consequence, **d** shows increased switching frequencies within all of the four PSG stages. Importantly, the relative differences between the PSG stages were effectively unchanged (even if the significant difference between 'Wake' and 'N2' was no longer evident). Interestingly, in **e**, the number of unique HMM states visited per unit time were numerically quite stable with or without the use of temporal filter.

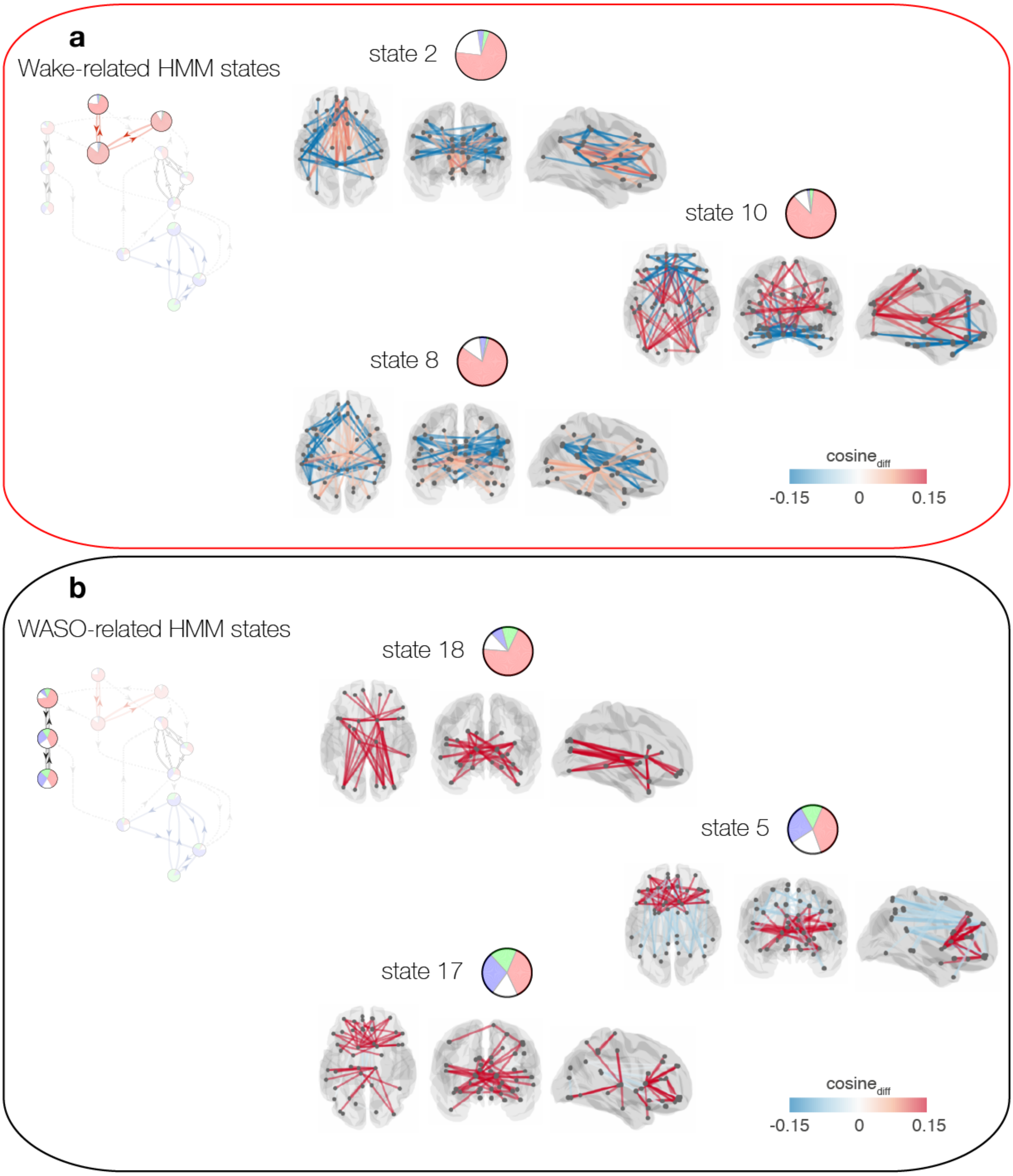

Supplementary Figure 17. ***Differential cosine similarity maps of wakefulness-related HMM states.*** (supplement to Supplementary Figure 4). Produced to show that the differential FC maps of Supplementary Figure 4 were not affected by the different mean activations of the HMM states. Please note the qualitative overlap with maps in Supplementary Figure 4. Like for Supplementary Figure 4, the maps show the 1% most negative and 1% most positive weights.

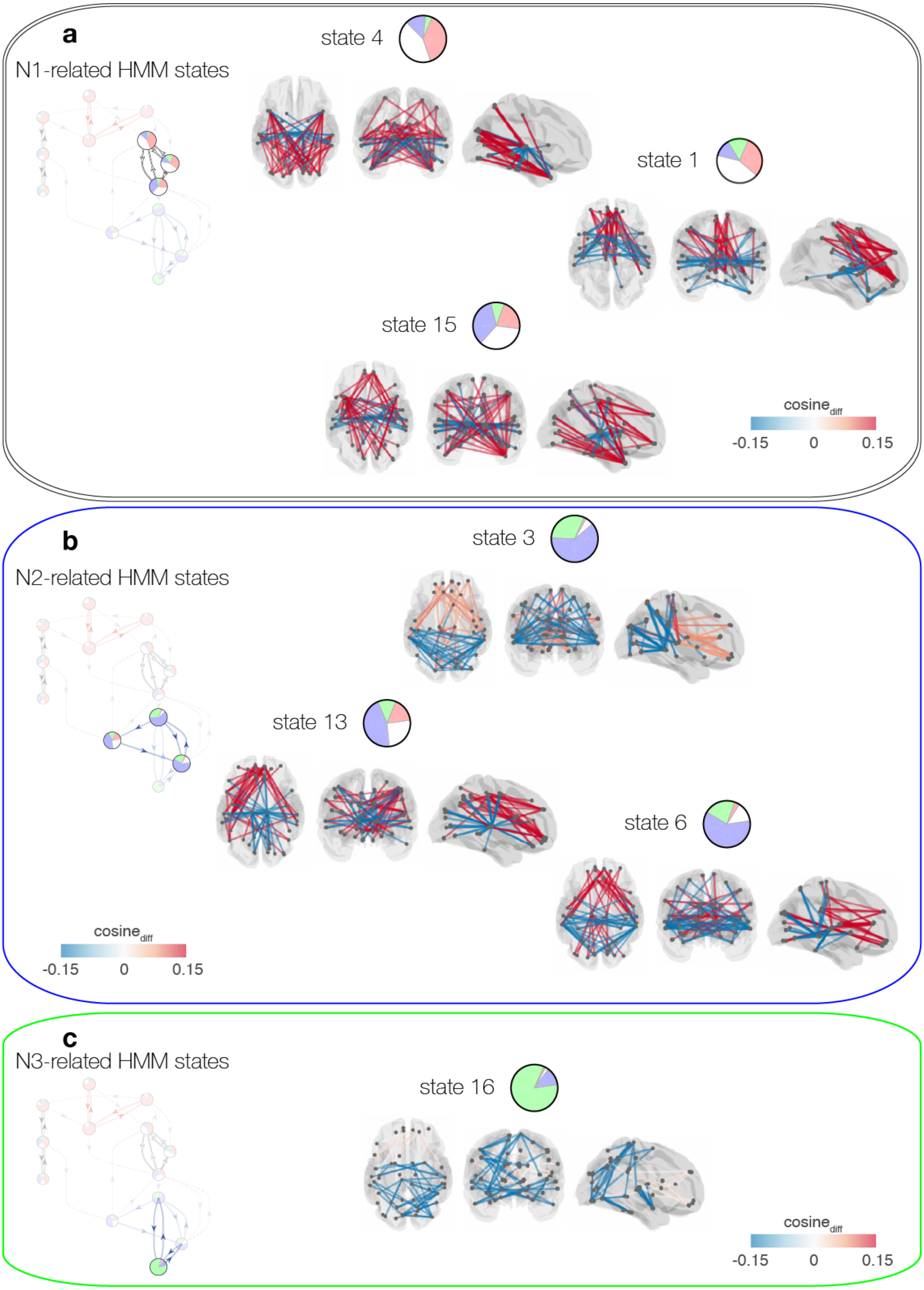
a
N1-related HMM states
state 4
state 1
state 15
cosine diff
-0.15
0
0.15
b
N2-related HMM states
state 3
state 13
state 6
cosine diff
-0.15
0
0.15
c
N3-related HMM states
state 16
cosine diff
-0.15
0
0.15

Supplementary Figure 18. ***Differential cosine similarity maps of wakefulness-related HMM states.*** (supplement to Supplementary Figure 5). Produced to show that the differential FC maps of Supplementary Figure 5 were not affected by the different mean activations of the HMM states. Please note the qualitative overlap with maps in Supplementary Figure 5. Like for Supplementary Figure 5, the maps show the 1% most negative and 1% most positive weights.

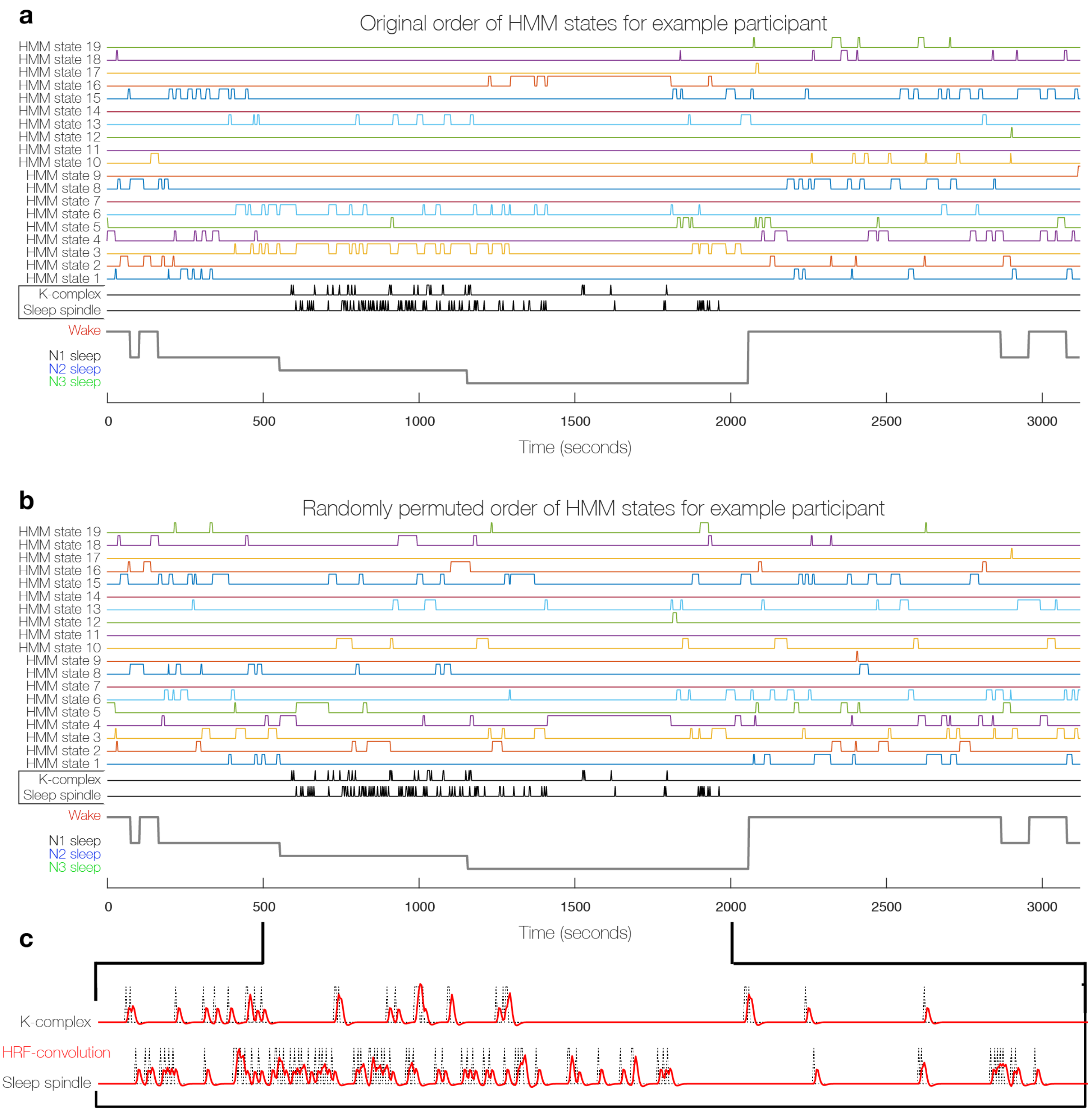

Supplementary Figure 19. ***Presence of sleep graphoelements in example participant.*** **a** Plotted together are the 19 HMM state timecourses, the markers of sleep spindles and K-complexes (SS-timecourse and KC-timecourse), and the PSG scoring for an example participant. The SS- and KC-timecourses were based on AASM scoring of sleep graphoelements in the EEG data. Specifically, SS-timecourse and KC-timecourse were binary and of the same length as the fMRI data, with ones representing the fMRI samples during which the respective graphoelement occurred. **b** The same information for the same participant is plotted again, however this time the HMM state timecourses have been randomly permuted. Each permutation consisted in a random switching of the labels of each instance of an HMM state, keeping the number of occurrences of each HMM state and state transition times constant within participants. This was done 1000 times for the purpose of comparing correlation, sensitivity, and specificity of the HMM states to the presence of sleep graphoelements (see Supplementary Figures 20 and 21). **c** The analyses were also performed after convolution of the SS- and KC-timecourses with the canonical hemodynamic response function (HRF). An illustration of the convolution is shown in an enlarged view of the SS- and KC-timecourses from ~500 to ~ 2000 seconds. The red timecourses with characteristic delays and undershoots represent the HRF-convoluted SS- and KC-timecourses for this example participant.

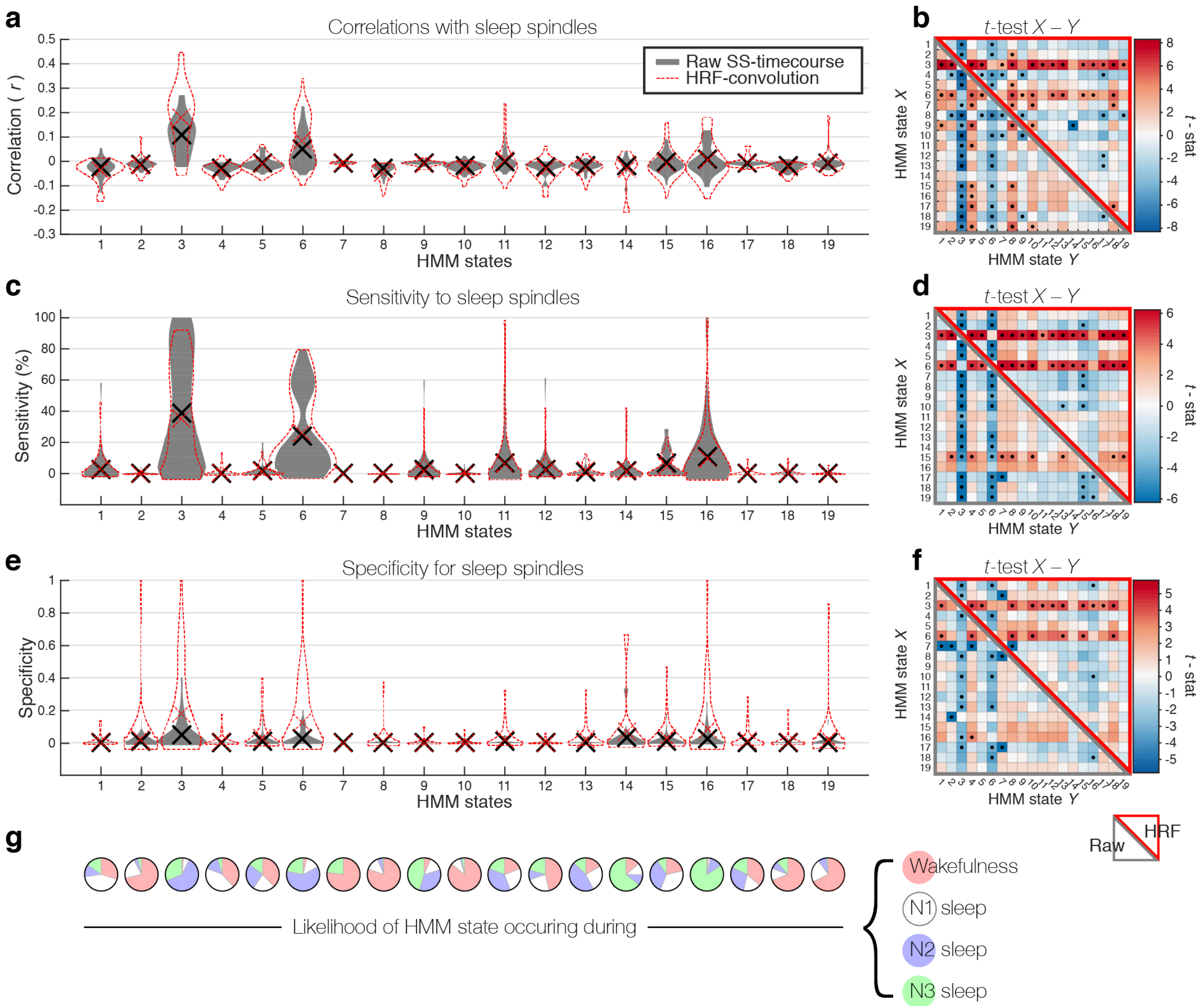

Supplementary Figure 20. ***Relationship between the 19 HMM states and the presence of sleep spindles.*** **a** The grey violin plot shows the distribution of Pearson's correlation values computed between the timecourse of each HMM state and the raw timecourse of sleep spindles for each of the 57 participants that included sleep spindles (see Supplementary Table 3 for summary statistics of sleep spindles). The black crosses denote the means across participants. In red are shown the outlines of an equivalent violin plot when considering HRF-convolved versions of the spindle timecourses. **b** The sub-diagonal part of the 19×19 matrix includes the *t*-statistics resulting from paired *t*-tests on the correlation values between each pair of the 19 HMM states. The super-diagonal part includes the *t*-stats considering the HRF-convolved versions of the spindle timecourses. A black star in the center of an entry (x, y) denotes a significant difference between the corresponding pair of HMM state X and Y as evaluated through 1000 random permutations of the HMM state timecourses, at a significance level that has been Bonferroni-corrected for the multiple comparisons between pairs of HMM states. **c** Distributions of sensitivity of each of the 19 HMM states to sleep spindles across participants for raw spindle timecourses (grey) and HRF convolved spindles (red). Sensitivity was defined as the proportion of sleep spindles that occurred within a given HMM state. **d** Equivalent to **b** but for sensitivity values. **e** Distributions of the 19 HMM states' specificity for sleep spindles across participants for raw spindle timecourses (grey) and HRF convolved spindles (red). Specificity was defined as the likelihood of finding a given HMM state active during a spindle, i.e. the ratio of an HMM state's occurrences taking place during spindles. **f** Equivalent to **b** and **d** but for specificity values. **g** For reference are included the circle plots, used throughout the manuscript, indicating the specificity of each HMM state to the sleep stages, calculated for the 18 participants that included all sleep stages. It is clear that sleep spindles correlated higher with the HMM states with high specificity for N2 sleep. HMM states 3 and 6 were thus found to correlate

significantly higher with spindles than most of the other HMM states, while no significant difference were found between the two. This was true regardless of HRF convolution of the spindles. HMM states 3 and 6 also accounted for the majority of spindle occurrences, as quantified through their sensitivity. Given the generally low specificity values, it is also clear that no HMM state occurred exclusively during spindles.

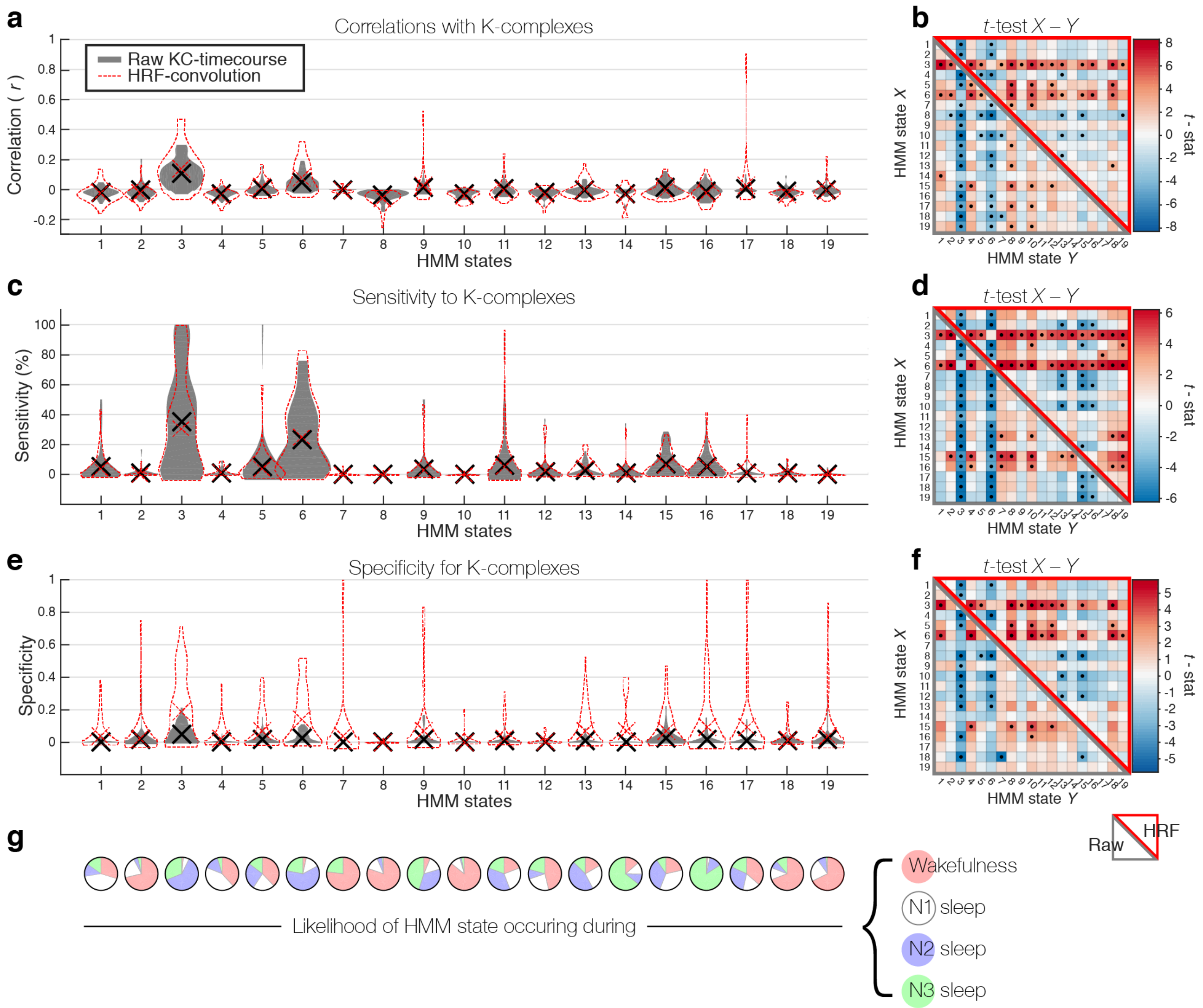

Supplementary Figure 21. ***Relationship between the 19 HMM states and the presence of K-complexes.*** **a** The grey violin plot shows the distribution of Pearson's correlation values computed between the timecourse of each HMM state and the raw timecourse of K-complexes for each of the 57 participants that included K-complexes (see Supplementary Table 3 for summary statistics of K-complexes). The black crosses denote the means across participants. In red are shown the outlines of an equivalent violin plot when considering HRF-convolved versions of the KC-timecourses. **b** The sub-diagonal part of the 19×19 matrix includes the *t*-statistics resulting from paired *t*-tests on the correlation values between each pair of the 19 HMM states. The super-diagonal part includes the *t*-stats considering the HRF-convolved versions of the KC-timecourses. A black star in the center of an entry (x, y) denotes a significant difference between the corresponding pair of HMM state X and Y as evaluated through 1000 random permutations of the HMM state timecourses, at a significance level that has been Bonferroni-corrected for the multiple comparisons between pairs of HMM states. **c** Distributions of sensitivity of each of the 19 HMM states to K-complexes across participants for raw KC-timecourses (grey) and HRF convolved K-complexes (red). Sensitivity was defined as the proportion of K-complexes that occurred within a given HMM state. **d** Equivalent to **b** but for sensitivity values. **e** Distributions of the 19 HMM states' specificity for K-complexes across participants for raw KC-timecourses (grey) and HRF convolved K-complexes (red). Specificity was defined as the likelihood of finding a given HMM state active during a K-complex, i.e. the ratio of an HMM state's occurrences taking place during K-complexes. **f** Equivalent to **b** and **d** but for specificity values. **g** For reference are included the circle plots, used throughout the manuscript, indicating the specificity of each HMM state to the sleep stages, calculated for the 18 participants that included all sleep stages. It is clear that the HMM states relate to K-complexes in a fashion highly similar to that of spindles, presented in Supplementary Figure 20. K-complexes correlated higher with the HMM states with high specificity for N2 sleep. HMM states 3 and 6 were thus found to

correlate significantly higher with K-complexes than most of other HMM states, while no significant difference were found between the two. HMM states 3 and 6 also accounted for the majority of K-complex occurrences, as quantified through their sensitivity. Given the generally low specificity values, it is also clear that no HMM state occurred exclusively during K-complexes.

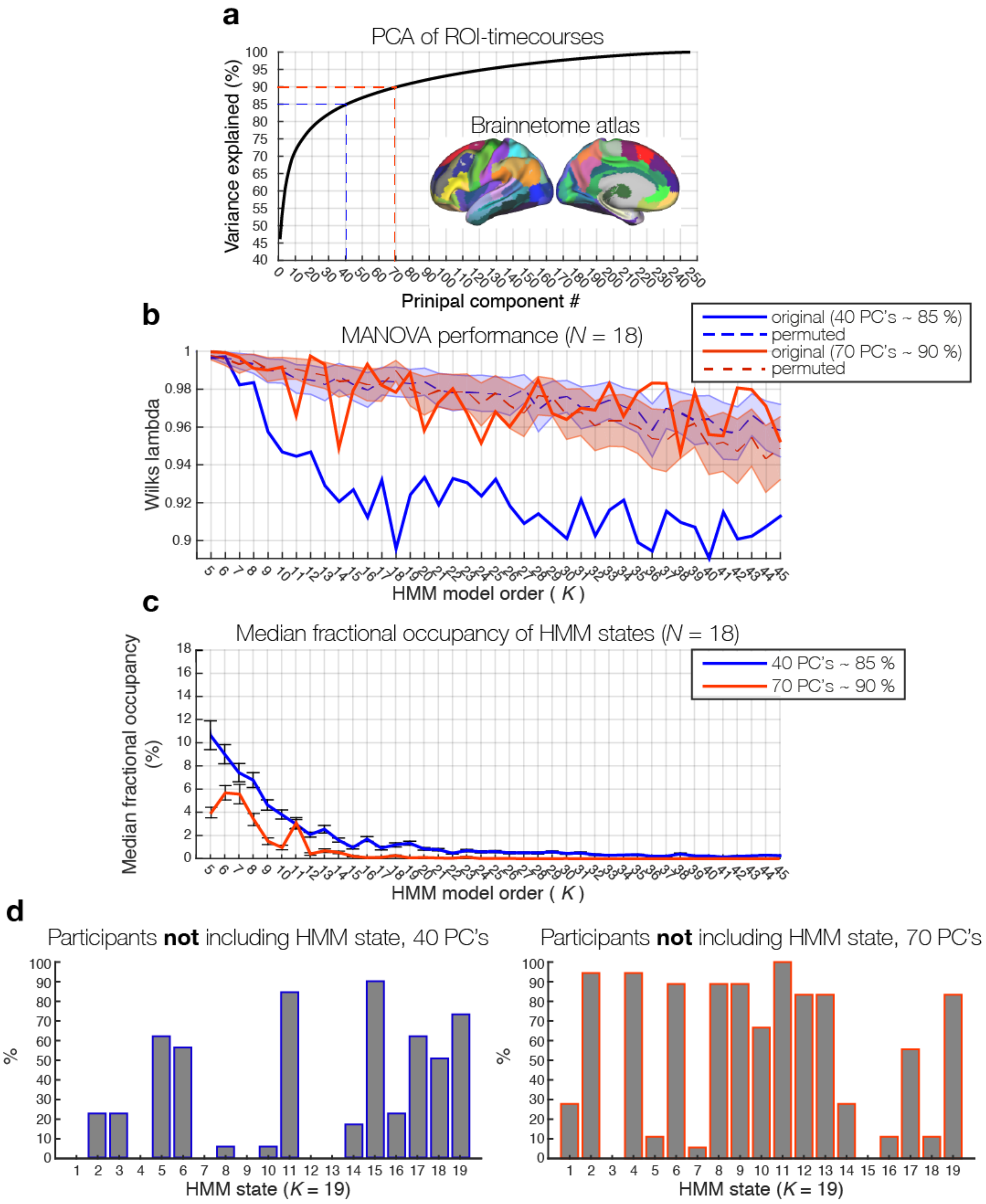

Supplementary Figure 22. ***Performance of HMM using the Brainnetome atlas.*** **a** Curve showing the cumulative percentage of variance represented by the components of the PCA performed on the ROI timecourses extracted from the Brainnetome atlas (see Figure 1b for an equivalent plot for the AAL data). The blue and the red dashed lines show the two cases analysed; 40 PC's ~ 85% and 70 PC's ~ 90%, respectively. **b** Plot showing the development across HMM model orders (from 5 to 45 states) of the MANOVA performance of the HMM when compared to the EEG-based sleep scoring for the 18 participants that included all PSG stages (see Supplementary Figure 1b for an equivalent plot for the AAL data). Going

from 90% of the variance (red) to 85% of the variance (blue) had a significant effect on how well the HMM states related to the PSG scoring. Notice how the red line rarely goes below the zone representing the permuted, random cases, whereas the blue line emulates the original analysis on the AAL data (see curve in Supplementary Figure 1b). **c** Tracking of the median fractional occupancy of the HMM states across model orders, within the 18 participants that included all four PSG stages. In blue is shown the curve for 40 PC's ~ 85%, while the result using 70 PC's ~ 90% is shown in red (An equivalent plot for the AAL data may be found in Supplementary Figure 1c). The fact that the red line is consistently lower than the blue suggests that using the higher percentage of variance implied a high occurrence of 'sporadic' HMM states that accounted for only small portions of the data. This is also evident in **d** where the HMM solution using 19 states are shown in more detail, for 40 PC's on the left and for 70 PC's on the right. These plots are equivalent to that of Supplementary Figure 6b, which pertains to the original HMM on the AAL data, and show the percentage of participants that did not include each of the 19 HMM states. Using 40 PC's ~ 85 % of variance produced a result more similar to the original HMM on the AAL, with 12 HMM states being included in more than 25% of the participants, whereas including 70 PC's ~ 90% meant that only 6 HMM states were represented in more than 25% of the participants. Error bars in **b** and **c** represent standard error across HMM states within a model order.

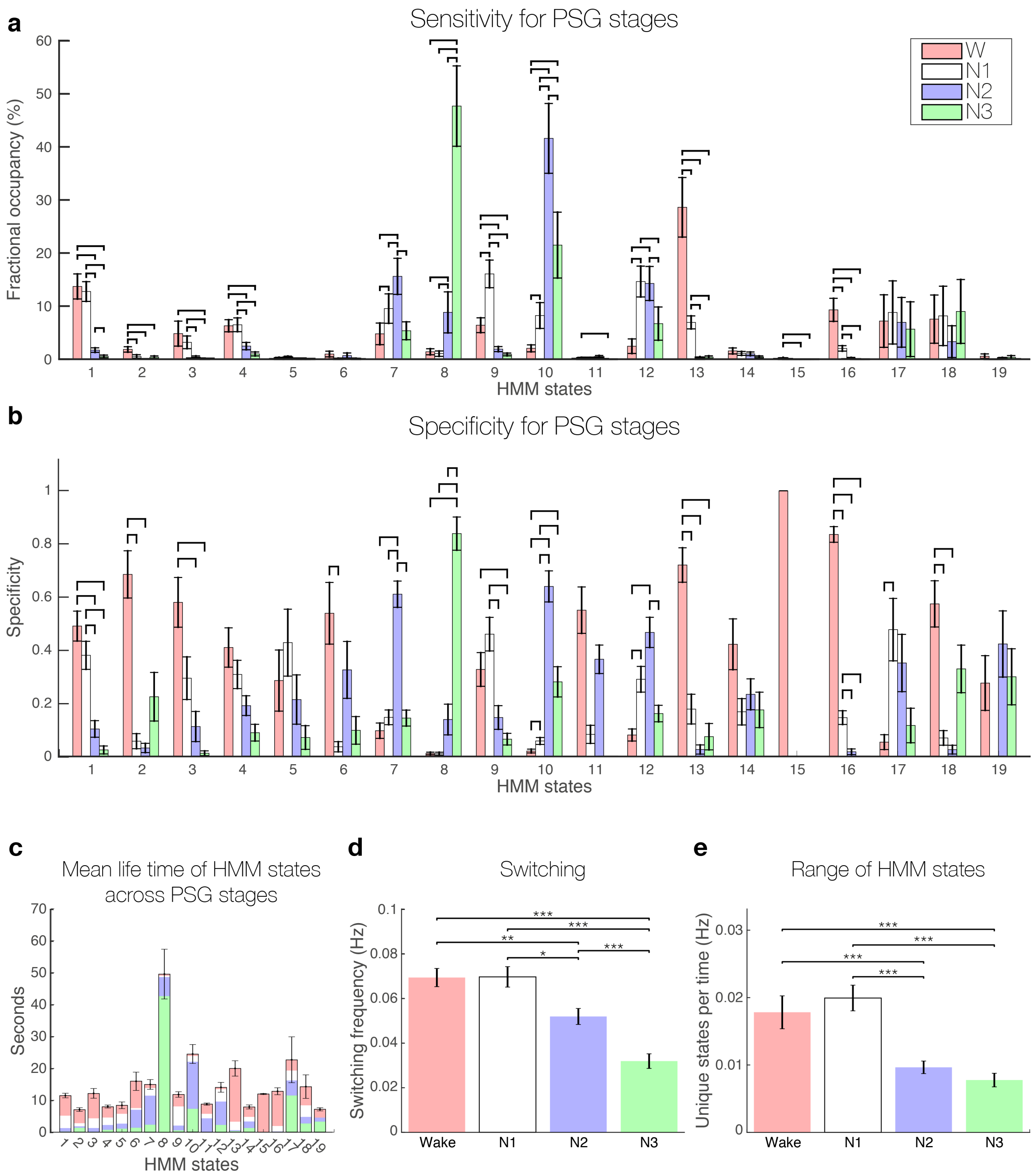

Supplementary Figure 23. ***Robustness of HMM results when using an alternative parcellation (Brainnetome)*** (Equivalent to Figure 3 but for the HMM run on 40 PC's ~ 85% of the variance of the Brainnetome ROI timecourses with 19 states). **a** Select HMM states account for the majority of different PSG stages as quantified through their fractional occupancies. **b** In the same way as for the original analysis on the AAL data there is an overlap between the HMM states with high sensitivity for a given PSG stage and the HMM states with high specificity for the same PSG stage. **c** HMM states with high specificity for N3 sleep expressed higher mean life times, as was the case for the original analysis. **d** The relative as well as the absolute values of switching were very similar to those of the original analysis on the AAL data. **e** Similarly to the switching dynamics in E, the ranges of unique HMM states visited within each PSG stage were similar to the original analysis. Please note that all plots were calculated from the 18 participants that included all

PSG stages, and that significant differences between HMM states or PSG stages were calculated in the same way as for Figure 3.

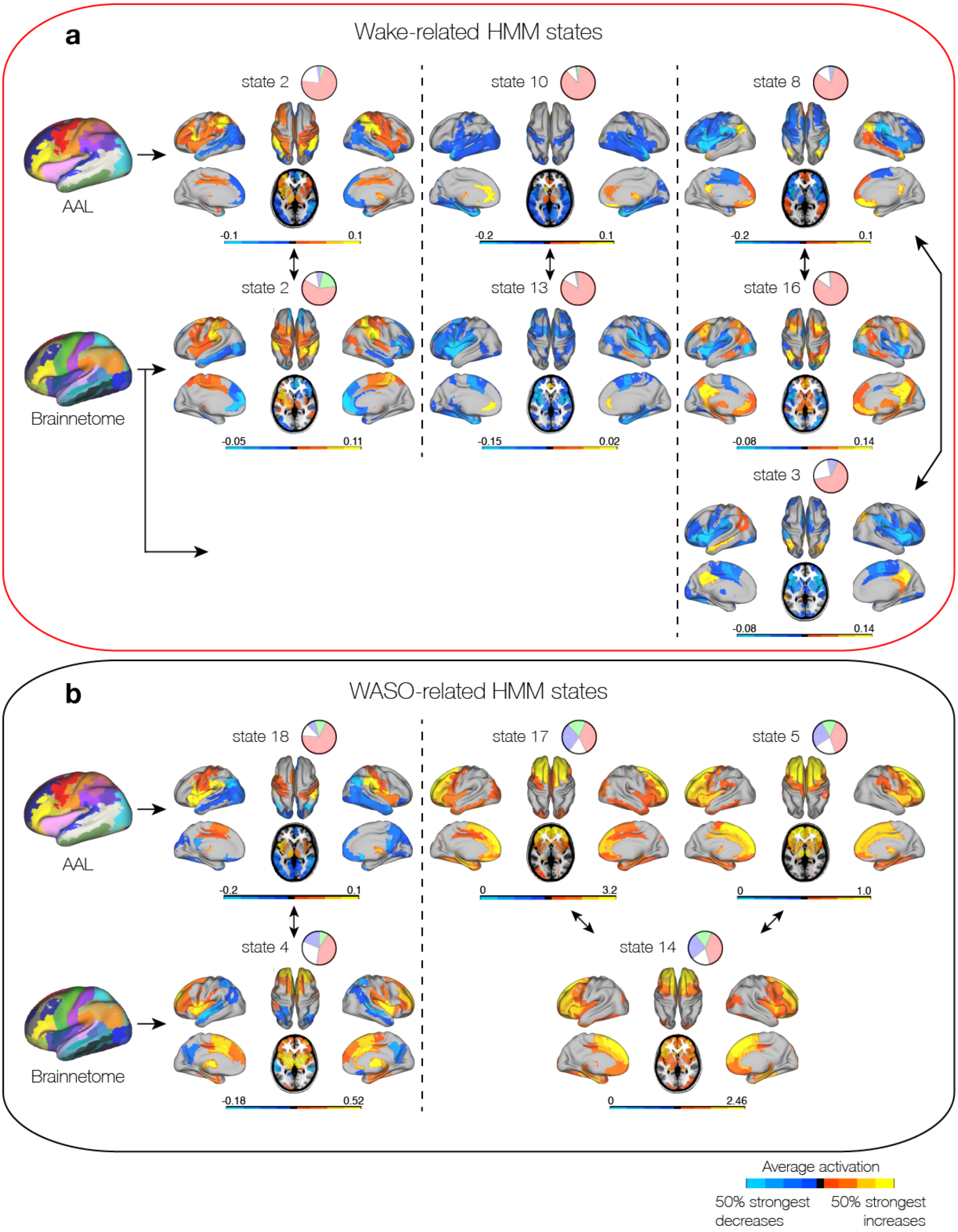

Supplementary Figure 24. ***Spatial correspondence between HMM states from AAL and Brainnetome in wakefulness-related HMM states.*** (Brain plots from the Brainnetome data are extracted from the HMM solution with 19 states on the 40 PC's ~ 85% of the variance). To demonstrate the correspondence between the original HMM solution on the AAL data and the HMM solution on the Brainnetome data, the original brain plots of mean activation distributions (from Figure 5) are shown together with brain plots from the

HMM on Brainnetome. These have been matched based on visual similarity between the spatial maps and their specificity profiles for PSG stages, represented in pie plots. **a** The original wake-related HMM states from the AAL together with HMM states from the Brainnetome. Notice the high correspondence not only in spatial distribution but also in the PSG-specificity. The original HMM state 8 appeared to show similarity to two HMM states from the Brainnetome analysis (HMM states 16 and 3). **b** The original three WASO-related HMM states appeared to have two equivalents in the Brainnetome solution.

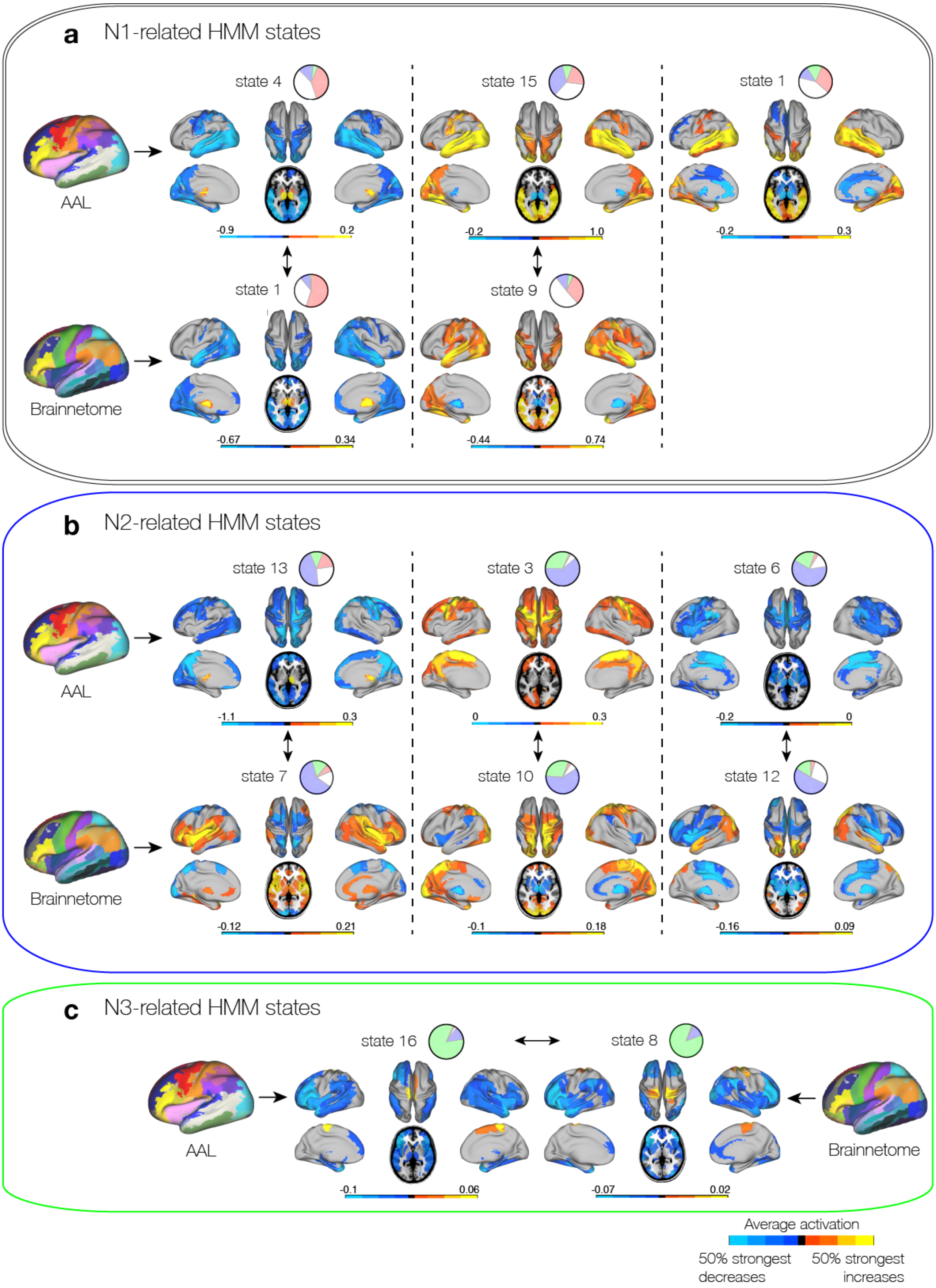

Supplementary Figure 25. ***Spatial correspondence between HMM states from AAL and Brainnetome in N1-, N2-, and N3-related HMM states.*** (Equivalent to Supplementary Figure 24, but for N1-, N2-, and N3-

sleep) **A)** The original N1-related HMM states found two equivalents from the Brainnetome HMM states. **B)** Each of the three original N2-related HMM states had equivalents from the Brainnetome HMM, which was also the case for the original N3-related HMM state shown in **C**.

Supplementary References

1. Maquet P. Functional neuroimaging of normal human sleep by positron emission tomography. *Journal of sleep research* **9**, 207-231 (2000).

2. Hofle N*, et al.* Regional Cerebral Blood Flow Changes as a Function of Delta and Spindle Activity during Slow Wave Sleep in Humans. *The Journal of Neuroscience* **17**, 4800-4808 (1997).

3. Braun AR*, et al.* Regional cerebral blood flow throughout the sleep-wake cycle. An H2(15)O PET study. *Brain : a journal of neurology* **120 (Pt 7)**, 1173-1197 (1997).

4. Kjaer TW, Law I, Wiltschiøtz G, Paulson OB, Madsen PL. Regional cerebral blood flow during light sleep–a H215O - PET study. *J Sleep Res* **11**, 201-207 (2002).

5. Laufs H*, et al.* Where the BOLD signal goes when alpha EEG leaves. *NeuroImage* **31**, 1408-1418 (2006).

6. Horovitz SG*, et al.* Low frequency BOLD fluctuations during resting wakefulness and light sleep: a simultaneous EEG-fMRI study. *Hum Brain Mapp* **29**, 671-682 (2008).

7. Dang-Vu TT*, et al.* Spontaneous neural activity during human slow wave sleep. *Proceedings of the National Academy of Sciences of the United States of America* **105**, 15160-15165 (2008).

8. Brodbeck V*, et al.* EEG microstates of wakefulness and NREM sleep. *NeuroImage* **62**, 2129-2139 (2012).

9. Tagliazucchi E, Laufs H. Decoding wakefulness levels from typical fMRI resting-state data reveals reliable drifts between wakefulness and sleep. *Neuron* **82**, 695-708 (2014).

10. Tagliazucchi E, von Wegner F, Morzelewski A, Borisov S, Jahnke K, Laufs H. Automatic sleep staging using fMRI functional connectivity data. *NeuroImage* **63**, 63-72 (2012).

11. Altmann A*, et al.* Validation of non-REM sleep stage decoding from resting state fMRI using linear support vector machines. *NeuroImage* **125**, 544-555 (2016).

12. Haimovici A, Tagliazucchi E, Balenzuela P, Laufs H. On wakefulness fluctuations as a source of BOLD functional connectivity dynamics. *Sci Rep* **7**, 5908 (2017).

13. Horovitz SG*, et al.* Decoupling of the brain's default mode network during deep sleep. *Proceedings of the National Academy of Sciences of the United States of America* **106**, 11376-11381 (2009).

14. Larson-Prior LJ, Zempel JM, Nolan TS, Prior FW, Snyder AZ, Raichle ME. Cortical network functional connectivity in the descent to sleep. *Proceedings of the National Academy of Sciences of the United States of America* **106**, 4489-4494 (2009).

15. Sämann PG*, et al.* Development of the brain's default mode network from wakefulness to slow wave sleep. *Cerebral cortex* **21**, 2082-2093 (2011).

16. Tagliazucchi E, von Wegner F, Morzelewski A, Brodbeck V, Jahnke K, Laufs H. Breakdown of long-range temporal dependence in default mode and attention networks during deep sleep. *Proceedings of the National Academy of Sciences of the United States of America* **110**, 15419-15424 (2013).

17. Snyder AZ. Intrinsic brain activity and resting state networks. In: *Neuroscience in the 21st Century* (ed^(eds Pfaff DW, Volkow ND). Springer New York (2016).

18. Schabus M*, et al.* Hemodynamic cerebral correlates of sleep spindles during human non-rapid eye movement sleep. *Proceedings of the National Academy of Sciences of the United States of America* **104**, 13164-13169 (2007).

19. Andrade KC*, et al.* Sleep spindles and hippocampal functional connectivity in human NREM sleep. *The Journal of neuroscience : the official journal of the Society for Neuroscience* **31**, 10331-10339 (2011).

20. Caporro M*, et al.* Functional MRI of sleep spindles and K-complexes. *Clinical neurophysiology : official journal of the International Federation of Clinical Neurophysiology* **123**, 303-309 (2012).

21. Jahnke K*, et al.* To wake or not to wake? The two-sided nature of the human K-complex. *NeuroImage* **59**, 1631-1638 (2012).

22. Laufs H, Walker MC, Lund TE. 'Brain activation and hypothalamic functional connectivity during human non-rapid eye movement sleep: an EEG/fMRI study'--its limitations and an alternative approach. *Brain : a journal of neurology* **130**, e75; author reply e76 (2007).

23. Nir Y*, et al.* Regional slow waves and spindles in human sleep. *Neuron* **70**, 153-169 (2011).

24. Piantoni G, Halgren E, Cash SS. Spatiotemporal characteristics of sleep spindles depend on cortical location. *NeuroImage* **146**, 236-245 (2017).

25. Mak-McCully RA, *et al.* Distribution, Amplitude, Incidence, Co-Occurrence, and Propagation of Human K-Complexes in Focal Transcortical Recordings. *eNeuro* **2**, ENEURO.0028-0015.2015 (2015).

26. Maxim V, *et al.* Fractional Gaussian noise, functional MRI and Alzheimer's disease. *NeuroImage* **25**, 141-158 (2005).

27. He BJ. Scale-free properties of the functional magnetic resonance imaging signal during rest and task. *Journal of Neuroscience* **31**, 13786-13795 (2011).

28. Ciuciu P, Varoquaux G, Abry P, Sadaghiani S, Kleinschmidt A. Scale-Free and Multifractal Time Dynamics of fMRI Signals during Rest and Task. *Front Physiol* **3**, 186 (2012).

29. von Wegner F, Tagliazucchi E, Laufs H. Information-theoretical analysis of resting state EEG microstate sequences - non-Markovianity, non-stationarity and periodicities. *NeuroImage* **158**, 99-111 (2017).

30. Vidaurre D, Smith SM, Woolrich MW. Brain network dynamics are hierarchically organized in time. *Proceedings of the National Academy of Sciences of the United States of America* **114**, 12827-12832 (2017).

31. Allen EA, Damaraju E, Plis SM, Erhardt EB, Eichele T, Calhoun VD. Tracking whole-brain connectivity dynamics in the resting state. *Cerebral cortex (New York, NY : 1991)* **24**, 663-676 (2014).

32. Hindriks R, *et al.* Can sliding-window correlations reveal dynamic functional connectivity in resting-state fMRI? *NeuroImage* **127**, 242-256 (2015).

33. Tagliazucchi E, Balenzuela P, Fraiman D, Chialvo DR. Criticality in large-scale brain FMRI dynamics unveiled by a novel point process analysis. *Frontiers in physiology* **3**, 15 (2012).

34. Liu X, Duyn JH. Time-varying functional network information extracted from brief instances of spontaneous brain activity. *Proceedings of the National Academy of Sciences* **110**, 4392-4397 (2013).

35. Karahanoğlu FI, Ville DVD. Transient brain activity disentangles fMRI resting-state dynamics in terms of spatially and temporally overlapping networks. *Nature communications* **6**, 7751 (2015).

36. Calhoun Vince D, Miller R, Pearlson G, Adalı T. The Chronnectome: Time-Varying Connectivity Networks as the Next Frontier in fMRI Data Discovery. *Neuron* **84**, 262-274 (2014).

37. Preti MG, Bolton TAW, Ville DVD. The dynamic functional connectome: State-of-the-art and perspectives. *NeuroImage*, (2016).

38. Baker AP*, et al.* Fast transient networks in spontaneous human brain activity. *eLife* **3**, e01867 (2014).

39. Vidaurre D, Quinn AJ, Baker AP, Dupret D, Tejero-Cantero A, Woolrich MW. Spectrally resolved fast transient brain states in electrophysiological data. *NeuroImage* **126**, 81-95 (2016).

40. Vidaurre D*, et al.* Spontaneous cortical activity transiently organises into frequency specific phase-coupling networks. *Nature communications* **9**, 2987 (2018).

41. Ryali S*, et al.* Temporal Dynamics and Developmental Maturation of Salience, Default and Central-Executive Network Interactions Revealed by Variational Bayes Hidden Markov Modeling. *PLoS computational biology* **12**, e1005138 (2016).

42. Deco G*, et al.* Perturbation of whole-brain dynamics in silico reveals mechanistic differences between brain states. *NeuroImage* **169**, 46-56 (2018).

43. Jobst BM*, et al.* Increased Stability and Breakdown of Brain Effective Connectivity During Slow-Wave Sleep: Mechanistic Insights from Whole-Brain Computational Modelling. *Sci Rep* **7**, 4634 (2017).

44. Deco G, Hagmann P, Hudetz AG, Tononi G. Modeling resting-state functional networks when the cortex falls asleep: local and global changes. *Cerebral cortex* **24**, 3180-3194 (2014).

45. Cabral J, Kringelbach ML, Deco G. Functional connectivity dynamically evolves on multiple time-scales over a static structural connectome: Models and mechanisms. *NeuroImage* **160**, 84-96 (2017).

46. Lavigne G, Kryger M. Chapter 11 - Relevance of Sleep Physiology for Sleep Medicine Clinicians. In: *Principles and Practice of Sleep Medicine (Sixth Edition)* (ed^(eds Kryger M, Roth T, Dement WC). Elsevier (2017).

47. Ogilvie RD, Wilkinson RT. The detection of sleep onset: behavioral and physiological convergence. *Psychophysiology* **21**, 510-520 (1984).

48. Bonnet MH, Moore SE. The threshold of sleep: perception of sleep as a function of time asleep and auditory threshold. *Sleep* **5**, 267-276 (1982).

49. Siclari F*, et al.* The neural correlates of dreaming. *Nature neuroscience* **20**, 872-878 (2017).

50. Nobili L*, et al.* Chapter 13 - Local aspects of sleep: Observations from intracerebral recordings in humans. In: *Progress in Brain Research* (ed^(eds Andries Kalsbeek MMTR, Russell GF). Elsevier (2012).

51. Goupil L, Bekinschtein TA. Cognitive processing during the transition to sleep. *Archives italiennes de biologie* **150**, 140-154 (2012).

52. Hori T, Hayashi M, Morikawa T. Topographical EEG changes and the hypnagogic experience. (1994).

53. Tassi P, Muzet A. Sleep inertia. *Sleep medicine reviews* **4**, 341-353 (2000).

54. Diekelmann S, Born J. The memory function of sleep. *Nature reviews Neuroscience* **11**, 114-126 (2010).

55. Wei Y*, et al.* Sleep Stage Transition Dynamics Reveal Specific Stage 2 Vulnerability in Insomnia. *Sleep* **40**, zsx117-zsx117 (2017).

56. Chang C*, et al.* Tracking brain arousal fluctuations with fMRI. *Proceedings of the National Academy of Sciences* **113**, 4518-4523 (2016).

57. Stickgold R. Sleep-dependent memory consolidation. *Nature* **437**, 1272-1278 (2005).

58. Edinger JD, Ulmer CS, Means MK. Sensitivity and Specificity of Polysomnographic Criteria for Defining Insomnia. *Journal of clinical sleep medicine : JCSM : official publication of the American Academy of Sleep Medicine* **9**, 481-491 (2013).

59. Manconi M*, et al.* Measuring the error in sleep estimation in normal subjects and in patients with insomnia. *J Sleep Res* **19**, 478-486 (2010).

60. Littner M*, et al.* Practice parameters for using polysomnography to evaluate insomnia: an update. *Sleep* **26**, 754-760 (2003).

61. Ni Mhuircheartaigh R, Warnaby C, Rogers R, Jbabdi S, Tracey I. Slow-wave activity saturation and thalamocortical isolation during propofol anesthesia in humans. *Sci Transl Med* **5**, 208ra148 (2013).

62. Uhrig L, Dehaene S, Jarraya B. Cerebral mechanisms of general anesthesia. *Annales francaises d'anesthesie et de reanimation* **33**, 72-82 (2014).

63. Barttfeld P, Uhrig L, Sitt JD, Sigman M, Jarraya B, Dehaene S. Signature of consciousness in the dynamics of resting-state brain activity. *Proceedings of the National Academy of Sciences of the United States of America* **112**, 887-892 (2015).

64. Carhart-Harris RL*, et al.* The entropic brain: a theory of conscious states informed by neuroimaging research with psychedelic drugs. *Frontiers in human neuroscience* **8**, 20 (2014).

65. Dehaene S, Charles L, King JR, Marti S. Toward a computational theory of conscious processing. *Current opinion in neurobiology* **25**, 76-84 (2014).

66. Vidaurre D*, et al.* Discovering dynamic brain networks from big data in rest and task. *NeuroImage*, (2017).

67. Olbrich S*, et al.* EEG-vigilance and BOLD effect during simultaneous EEG/fMRI measurement. *NeuroImage* **45**, 319-332 (2009).

68. Eickhoff SB, Yeo BTT, Genon S. Imaging-based parcellations of the human brain. *Nature Reviews Neuroscience*, 1 (2018).

69. Fan L*, et al.* The Human Brainnetome Atlas: A New Brain Atlas Based on Connectional Architecture. *Cereb Cortex* **26**, 3508-3526 (2016).

70. Bhattacharyya A. On a measure of divergence between two statistical populations defined by their probability distributions. *Bulletin of the Calcutta Mathematical Society* **35**, 99-109 (1943).

71. Munkres J. Algorithms for the Assignment and Transportation Problems. *Journal of the Society for Industrial and Applied Mathematics* **5**, 32-38 (1957).

72. Iber C, Ancoli-Israel S, Chesson A, Quan SF. The AASM manual for the scoring of sleep and associated events: rules, terminology and technical specifications. (ed^(eds). American Academy of Sleep Medicine Westchester, IL (2007).

73. Piantoni G, Halgren E, Cash SS. The Contribution of Thalamocortical Core and Matrix Pathways to Sleep Spindles. *Neural plasticity* **2016**, 3024342 (2016).

74. Luthi A. Sleep spindles: where they come from, what they do. *The Neuroscientist : a review journal bringing neurobiology, neurology and psychiatry* **20**, 243-256 (2014).

75. Murphy M, Riedner BA, Huber R, Massimini M, Ferrarelli F, Tononi G. Source modeling sleep slow waves. *Proceedings of the National Academy of Sciences* **106**, 1608-1613 (2009).
